# Unveiling the fibrotic puzzle of endometriosis: An overlooked concern calling for prompt action

**DOI:** 10.12688/f1000research.152368.2

**Published:** 2024-10-02

**Authors:** Megha M Anchan, Guruprasad Kalthur, Ratul Datta, Kabita Majumdar, Karthikeyan P, Rahul Dutta

**Affiliations:** 1 Division of Reproductive Biology, Department of Reproductive Science, Kasturba Medical College, Manipal, Manipal Academy of Higher Education, Manipal, Karnataka, 576104, India; 2 Nova IVF fertility, Guwahati, Assam, India; 3 Gauhati Medical College & Hospital IVF centre, Bhangagarh, Gauhati Medical College, Assam, 781032, India; 4 Department of General Surgery, Government Kallakurichi Medical College, Government Kallakurichi Medical College, Kallakurichi, Tamil Nadu, India

**Keywords:** Endometriosis, pelvic pain, etiology, animal model, Epithelial-mesenchymal transition, fibrosis

## Abstract

Endometriosis is a benign, estrogen-dependent, persistent chronic inflammatory heterogeneous condition that features adhesions caused by estrogen-dependent periodic bleeding. It is characterised by a widely spread fibrotic interstitium that comprising of fibroblasts, myofibroblasts, collagen fibres, extracellular proteins, inflammatory cells, and active angiogenesis found outside the uterus. Thus, fibrosis is recognized as a critical component because of which current treatments, such as hormonal therapy and surgical excision of lesions are largely ineffective with severe side effects, high recurrence rates, and significant morbidity. The symptoms include dysmenorrhea (cyclic or non-cyclic), dyspareunia, abdominal discomfort, and infertility. The significant lack of knowledge regarding the underlying root cause, etiology, and complex pathogenesis of this debilitating condition, makes it challenging to diagnose early and to implement therapeutic approaches with minimal side effects presenting substantial hurdles in endometriosis management. Research on understanding the pathogenesis of endometriosis is still ongoing to find biomarkers and develop non-hormonal therapeutic approaches. Current clinical research indicates a close relationship between endometriosis and fibrosis, which is thought to be tightly linked to pain, a major factor for the decline in the patient’s quality of life but little is known about the underlying pathophysiological cellular and molecular signaling pathways that lead to endometriosis-related fibrosis. The available experimental disease models have tremendous challenges in reproducing the human characteristics of the disease to assess treatment effectiveness. Future translational research on the topic has been hindered by the lack of an adequate fibrotic model of endometriosis emphasizing the necessity of etiological exploration. This review article’s goal is to examine recent developments in the field and pinpoint knowledge gaps that exist with a focus on the development of novel fibrotic mouse models for the early diagnosis and treatment of endometriosis and how this knowledge aids in the development of novel anti-fibrotic treatments which opens fresh avenues for a thorough investigation and extended research in the field of endometriosis.

## Introduction

Endometriosis is an estrogen-dependent chronic inflammatory disorder resulting from the implantation of viable endometrial, epithelial, and stromal cells (lesions) outside the uterus and is often associated with infertility. ^1^ The condition affects at least 10% (~247 million) of women worldwide, with Asian women reporting the highest prevalence, with over ~42 million girls and women from India, ^2^^,^^3^ which can negatively affect the outcome of IVF treatments. ^4^^,^^5^ Endometriosis can result in severe dysmenorrhea, dyspareunia, and menorrhagia; exacerbates pelvic/abdominal pain; and eventually leads to infertility due to considerable damage to the structure and function of reproductive organs, even compromising the entire body system through the accumulation of fibrotic tissue. ^6^ The diagnosis can take 4 to 11 years due to difficulties in classifying and identifying the disease and its peculiar symptoms, as well as a lack of diagnostic indicators. ^7^ According to Maddern et al., endometriosis has a significant effect on a person’s quality of life, reproductive health, and society at large. ^8^ Currently, the most widely recognized theory explaining how endometriosis begins is “Sampson’s theory”, which holds that the misplaced viable endometrium-like tissue is transferred onto the pelvic peritoneum by retrograde menstruation via the fallopian tubes. ^9^ Even after several decades of research, the etiology is still unclear and depends on a few key theories and assumptions, such as retrograde menstruation theory, embryonic remnants, coelomic metaplasia, immune dysfunction, inflammation, oxidative stress, hormones, dysfunctional apoptosis, the microbiome, metabolomics, endocrinology, and genetic expression differences, which fail to adequately explain its pathophysiology. ^2^^,^^9^ Retrograde menstruation is prevalent in healthy women, and only a small population of women develop this condition, contributing to the understanding of complex mechanisms that underlie the onset of this challenging condition. ^10^ While 90% of women of reproductive age undergo retrograde menstruation to the pelvic cavity, only 10% of them develop endometriosis. These findings suggest that the onset and progression of the disease in the peritoneal cavity depend on additional relevant factors. ^11^ This entails understanding how cells from the normal lining of the uterus find atypical locations, multiply excessively, escape immune and apoptotic processes, and acquire the necessary blood supply and nutrients that ultimately result in the formation of aberrant fibrotic lesions that contribute to the distinctive symptoms triggered by endometriosis, including excruciating pain and infertility. ^12^ None of the available theories fully capture the intricacies of fibrotic endometriosis, emphasizing the need for additional studies to identify the pathophysiology of endometriosis. ^13^ EMT is a process in which epithelial cells lose the polarized structure of the cytoskeleton and acquire the enhanced motility of mesenchymal cells. These modifications are considered necessary for the original formation of endometriotic lesions. ^14^ While fibrosis has been recognized as a prominent component of endometriosis, its importance is underexplored, particularly in relation to EMT. The production of fibrotic tissue, which contains fibroblasts, myofibroblasts, collagen fibers, and inflammatory cells, is increasingly recognized as a crucial element contributing to disease severity, including resistance to treatment and high recurrence rates. ^15^ This paucity of understanding of the molecular and cellular mechanisms encouraging fibrotic endometriosis, particularly through processes such as EMT, provides an important barrier to the development of appropriate diagnostic tools and therapeutic strategies. Moreover, the American Society of Reproductive Medicine (rASRM) categorization score approach does not account for pathology-based staging on the basis of fibrosis, which includes EMT, the mesenchymal-to-epithelial transition (MET), or smooth muscle metaplasia (SMM). This means that patients with fibrotic characteristics and adhesions may fail to obtain a reliable diagnosis. ^16^ Integrating fibrosis-specific indicators into diagnostic standards should increase the reliability of endometriosis diagnosis and staging, allowing for more targeted and successful treatment options. ^17^ The formation, invasion, and angiogenesis of fibrotic ectopic lesions are associated with disrupted immunoregulatory processes and a variety of inflammatory markers, including immune cells, cytokines, chemokines, matrix metalloproteinases, and other components associated with the immune system. ^18^^,^^19^ This makes it imperative to investigate and characterize the molecules involved in the emergence of this disease. Thus, a thorough understanding of the mechanisms underlying the origin and evolution of endometriosis is crucial for managing and evaluating the risks associated with this condition. In this review, we intend to address these gaps by providing a detailed understanding of the role of fibrosis in endometriosis, with a special focus on EMT. We will evaluate existing models of endometriosis, identify significant research gaps, and propose new directions for exploration. By emphasizing the importance of fibrosis-induced EMT in the emergence of endometriosis, we aim to identify possibilities for the development of novel therapeutic options that target these processes, thus increasing the prognosis of patients.

## Method

We conducted an electronic database literature search of PubMed and Google Scholar for published research articles on endometriosis and endometriotic animal models. The search terms “endometriosis”, “endometriosis mouse model”, “primate model of endometriosis”, “endometriotic patients”, and “endometriosis-associated fibrosis” were used. Articles with thorough experimental data and definitive results were considered for inclusion; those with inconclusive research findings were eliminated. We incorporated clinical trials, surveys of endometriosis-affected women, and observational and experimental studies, including animal studies, as references. Research written in languages other than English was not considered. All the graphics were prepared via Biorender software ( BioRender.com).

## Literature review

### Fibrotic endometriosis overview: knowledge gaps and challenges

Endometriosis is characterized by the persistent occurrence of fibrosis and myofibroblasts within endometriotic lesions, which play critical roles in disease development, making fibrosis a molecular hallmark of endometriosis. ^15^ Notably, significant scarring is commonly linked to endometriosis. ^15^ Although the initial onset of endometriosis is associated with the existence of endometrial stroma and glands in abnormal locations, the endometrial components are often soon replaced by fibrotic and smooth muscle components. ^20^ For example, rectovaginal nodules frequently display glandular epithelium embedded deeply within fibromuscular tissue devoid of any surrounding stroma. ^21^ Similarly, in 40% of ovarian endometriomas, there is no detection of the endometrial epithelium, and the interior of the cyst is covered solely by fibrotic tissue ^22^ Despite being a crucial pathological feature of the disease, pelvic adhesions generally lack any endometrial components. ^23^ These adhesions contribute to the pathology of some common symptoms of endometriosis, including chronic pelvic pain, deep dyspareunia, and infertility presumably aggravated by these fibrotic formations. ^23^ The process by which endometriosis progresses to a malignant condition remains unknown. However, continuous inflammation, immunological dysregulation, and fibrosis, most likely caused by iron-induced oxidative stress, may lead to genetic changes, which may lead to malignant features. ^24^^,^^25^ Fibrosis is believed to be linked to pain, which is the disease’s most common symptom and the principal cause of a patient’s poor quality of life. ^26^ Thus, understanding the underlying mechanisms will help to understand why the morphological characteristics of the disease do not match the degree and nature of fibrosis-related pain reported. ^27^

Fibrotic tissue is characterized by excessive development of extracellular matrix (ECM) components inside and around inflamed or damaged tissue, and it is a typical and significant phase of tissue repair in all organs. Fibrosis involves activated platelets, macrophages, and myofibroblasts, which results in increased collagen deposition. ^28^ EMT is characterized by the transformation of polarized, stationary epithelial cells into highly motile mesenchymal cells. ^29^ Furthermore, fibrosis occurs with the transition from epithelial to mesenchymal cells in a variety of malignancies and is associated with poor prognosis. ^30^ Fibrosis and smooth muscle metaplasia are two of the main characteristics of endometriosis in women; fibrosis surrounds endometriotic tissue, and the degree of fibrosis is connected with the degree of smooth muscle metaplasia. ^31^ On the basis of these data, we postulate that fibrotic-based EMT involvement in chronic inflammatory responses may be a factor in the invasive nature of endometriotic lesions. Additionally, angiogenesis, which stimulates endothelial function, vascular permeability, and the emergence of experimental endometriosis, is commonly associated with increased invasive and metastatic potential. ^32^ Endometriotic lesions are thought to be “wounds” that undergo repeated tissue injury and repair (ReTIAR), leading to TGF-β1/Smad3-mediated EMT and ultimately resulting in fibrosis as the lesions progress. In essence, regardless of location or subtype, all endometriotic lesions are known to be similar to wounds that undergo ReTIAR, ultimately leading to the fibrotic emergence of both ovarian endometriomas (OMAs) and deep infiltrating endometriosis (DIE). From this viewpoint, comprehending the natural course of endometriotic lesions and recognizing the complexities involved in treating endometriosis are rather easy. ^25^^,^^33^ This makes it possible for solitary cells to pass through the basement membrane, grow invasively, and metastasize by both intra- and extravasation. Sampson’s implantation theory states that each of these occurrences is necessary for the development of an endometriotic lesion. ^34^

Owing to differences in opinions concerning the etiology of the disease, the EMT route has received less attention in the context of endometriosis than it does in cancer research. Recently, most research on EMT in endometriosis has focused on tissues; very few studies have examined the specific transcription factors involved in EMT signaling that are present in endometriotic cells ^35^^,^^36^ EMT-related processes in endometriosis have been reported to be far more prevalent in ectopic endometrial lesions than in eutopic endometria, suggesting that EMT may contribute to the development of endometriosis. ^37^ For example, in fibrosis of organs such as the lungs, liver, and kidney, the involvement of the TGF-β signaling pathway is well documented. ^38^ TGF-β is an influential growth factor and a chemical that attracts monocytes and is capable of triggering fibrosis and angiogenesis during abnormal growth and promoting the progression of endometriosis. ^39^ Compared with those of normal women, the peritoneal fluid of stage III and IV endometriosis patients has greater levels of TGF-β. ^40^ If the underlying mechanisms are unknown, they may explain why the disease’s morphological characteristics do not match the extent and nature of fibrosis-induced pain sensations. ^27^ Endometriosis research is mostly based on nonhuman primate or rodent models due to the apparent limitations and ethical concerns of human experimentation. The available mouse models have aided in investigating several aspects of the disorder, such as early disease phases, ^41^ steroid hormone involvement, ^42^ host inflammatory mechanisms, ^43^^,^^44^ oxidative stress, ^45^^,^^46^ neuroangiogenesis, ^47^ and infertility, ^48^ in mice. However, there is a paucity of information on the development of preclinical models that define clinically effective endpoints such as EMT-induced fibrosis. Consequently, studies on the molecular pathways associated with EMT or possible targets for therapeutic intervention for EMT and fibrosis in endometriosis have been stopped because of the unavailability of an animal model of endometriosis that mimics the human condition. Furthermore, 50–70% of drugs that have advanced to phase II and III clinical trials are unable to show efficacy, indicating the insufficiency of current disease models in the exploration of critical biological processes. ^49^ Additionally, in their mouse model of endometriosis, Modi et al. reported significant inflammation but no histological fibrosis, and no EMT or fibrosis is typical in endometriosis. ^50^ These findings suggest that there are not enough disease models to investigate crucial biological processes. Given the chronic nature of the disease, we anticipate that both EMT and fibrosis processes may play substantial roles in the evolution of endometriosis, potentially leading to fibrotic adenomyosis. In summary, we intend to highlight the need for an ideal model for investigating endometriosis that reflects the cellular and pathophysiological processes and clinical behaviors observed in human patients, specifically fibrosis leading to scar formation and EMT linked with invasion and metastasis.

### Endometriotic models: Importance of addressing gaps in preclinical animal models

**Figure 1.**
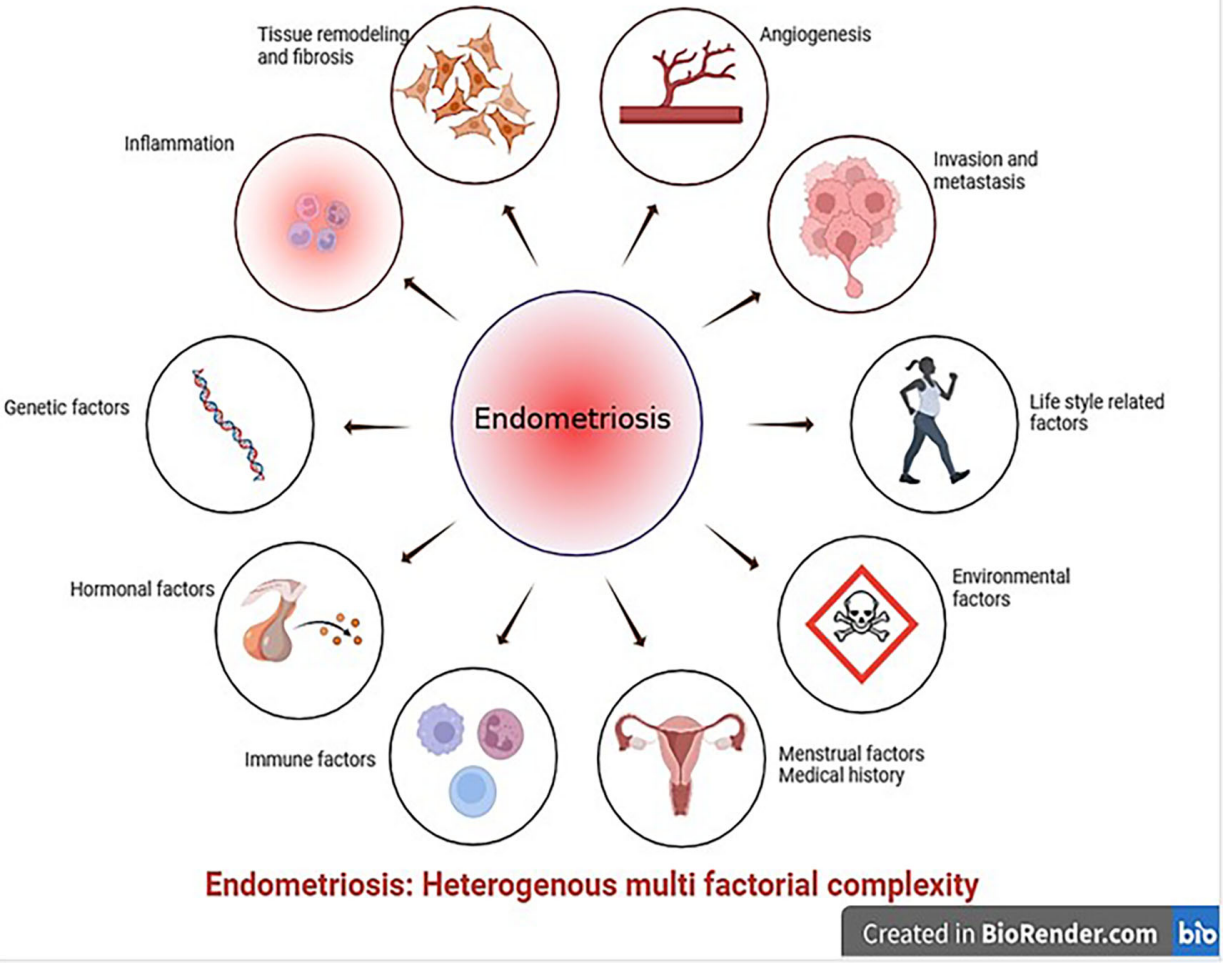
Schematic representation of the local triggers responsible for the development of endometriosis (created with Biorender.com).

Rodent models fail to accurately reproduce critical aspects of human endometriosis, such as EMT-induced chronic fibrosis. These limitations hamper the successful translation of research findings to human disease situations. This underlines the demand for a higher-fidelity mouse model that better depicts the complicated pathophysiology of endometriosis in humans. According to Greaves et al., endometriosis is currently being studied via two basic approaches: human-based *in vitro* samples and experimental *in vivo* animal models. The first type involves experimental *in vitro* research using tissue biopsies and fluids obtained from resected lesions or aspiration biopsies, such as endometrial and peritoneal explants, endometriotic cell lineages, primary endometrial stromal cells, endometrial stem cells, and immune cells. ^51^ *In vivo* animal models are essential for assessing drug candidates and for preclinical trial testing. Our knowledge of the early phases of disease development, including the effects of the peritoneal microenvironment, inflammatory responses, and steroid responsiveness, has improved because of these models. ^52^ However, replicating *in vitro* or *in vivo* models that accurately mimic the latent features identified in patients with endometriosis, such as EMT or fibrosis, is challenging for various reasons. First, endometriosis is complex, multifactorial, and heterogeneous, as none is certain condition’s onset or duration, making it challenging to model consistently. Second, the disease appears in numerous forms, including peritoneal, deep infiltrative lesions, and ovarian endometriomas, each displaying unique pathological features. ^53^ Finally, endometriosis cannot be effectively characterized on the basis of a single pathophysiological mechanism. Additionally, this condition is connected with genetic, ^54^ immunological, ^55^ environmental, ^56^^,^^57^ and hormonal changes, such as progesterone resistance ^58^ and estrogen reliance, ^59^ further challenging the establishment of acceptable animal models ( Figure 1).

### Limitations of available rodent models in endometriosis research

Endometriosis is termed the ‘missing disease’ because of its ambiguous etiology and discrepancies in its origin, diagnosis and treatment. ^60^ Despite a recent surge in endometriosis research, the underlying pathobiology of the disease remains poorly known, implying that animal models of the disorder are crucial for future studies in this field. This ambiguity highlights the need for animal models that precisely mimic human endometriosis and elucidate its conditions, which can provide a basis for subsequent research. ^61^ One of the most significant obstacles in endometriosis research is the lack of reliable mouse models that characterize the manifestations of this condition in humans. ^62^ Ideally, a disease model should mirror human disease while also allowing researchers to investigate the effects of intrinsic (e.g., genes) and extrinsic (e.g., environment) factors on disease progression. Many previous studies linked fibrosis secondary to the development of endometriosis, and there has not been much research on fibrosis itself. ^16^^,^^64^ Research from animal models clearly revealed that a percentage of women receiving hormone therapy in human trials do not respond to these drugs ^64^ and require surgical lesion removal to alleviate symptoms. Women may have endometriotic lesions that have progressed to a fibrotic state by the time they seek medical attention, rendering treatment ineffective. This highlights the urgent need for the establishment of an *in vivo* model that can effectively mimic the development and characteristics of human endometriosis, opening avenues for more effective treatments and a deeper understanding of this disease. These findings will also facilitate the understanding of the connection between the origin of fibrosis in endometriosis, existing medical care, and potential targets for therapy.

### Primate model of endometriosis

Until recently, the evidence supporting the process of fibrosis has been derived from *in vitro* experiments conducted in humans and from *in vivo* studies in more vertebrates, such as baboons. Nonhuman primates and higher vertebrates are potential candidates for disease research because of their anatomical resemblance to human reproductive organs. ^65^ Controlled experimental investigations on humans are limited because assessing disease prevalence and development necessitates numerous laparoscopies, which are challenging for a variety of reasons. Although endometriosis occurs spontaneously in humans, human investigations have been limited for ethical and practical reasons, with one of the primary reasons being the difficulty of studying the disease. As a result, understanding the mechanisms that cause this disease requires the use of an appropriate animal model. Endometriosis has long been investigated in both primates and nonprimate animals. The spontaneous endometriosis of the baboon ^66– 68^ limitation is that baboons have vast and effective mechanisms for clearing and regenerating their peritoneum. ^68^ In contrast, in rhesus monkeys, ^69^^,^^70^ the significance of peritoneal cysts in endometriosis pain and discomfort has not been investigated. The cynomolgus monkey, ^71^^,^^72^ has been described, with the limitations that deep lesions are difficult to diagnose and that time course changes in the condition are not investigated. Although nonhuman primates are excellent models for studying endometriosis, they are expensive to maintain and extremely sensitive to captivity. Furthermore, spontaneous endometriosis occurs at a low frequency, limiting the use of primates in research. ^73^ Although ideal regarding fidelity, the use of nonhuman primates in endometriosis research is restricted because they are expensive to maintain, require a longer duration to develop endometriotic lesions, spontaneous endometriosis occurs at a low frequency, an extended period of gestation for fertility research, and ethical aspects. ^74^ For numerous reasons, the mouse is increasingly adopted as a preclinical model development tool in biomedical research, as it is a molecularly well-annotated species that allows researchers to utilize several interrogative methods to explore complicated multifactorial diseases and is suitable for dissecting the molecular underpinnings of disease pathogenesis because of the simplicity of genetic modification and focused changes in candidate genes. ^74^ However, because research facilities for primates are restricted, nonprimate experimental animal species, such as mice or rats, are considered ideal first-line techniques for investigating the etiology of this mysterious disease.

### Rodent models of endometriosis

Every menstrual cycle, endometriosis is characterized by the development of new lesions and the advancement of preexisting lesions. Therefore, additional research is needed to understand the natural course and gradual development of endometriosis lesions. ^75^ There is evidence of gradual lesion clearing, but only a small number of studies using mouse models of endometriosis have investigated disease induction and regression ^75^^,^^76^ It is unethical to perform many laparoscopies on endometriosis patients to monitor disease progression. Therefore, longitudinal studies of lesion formation and progression can considerably increase the translational efficiency of preclinical models of endometriosis. ^75^

**Figure 2.**
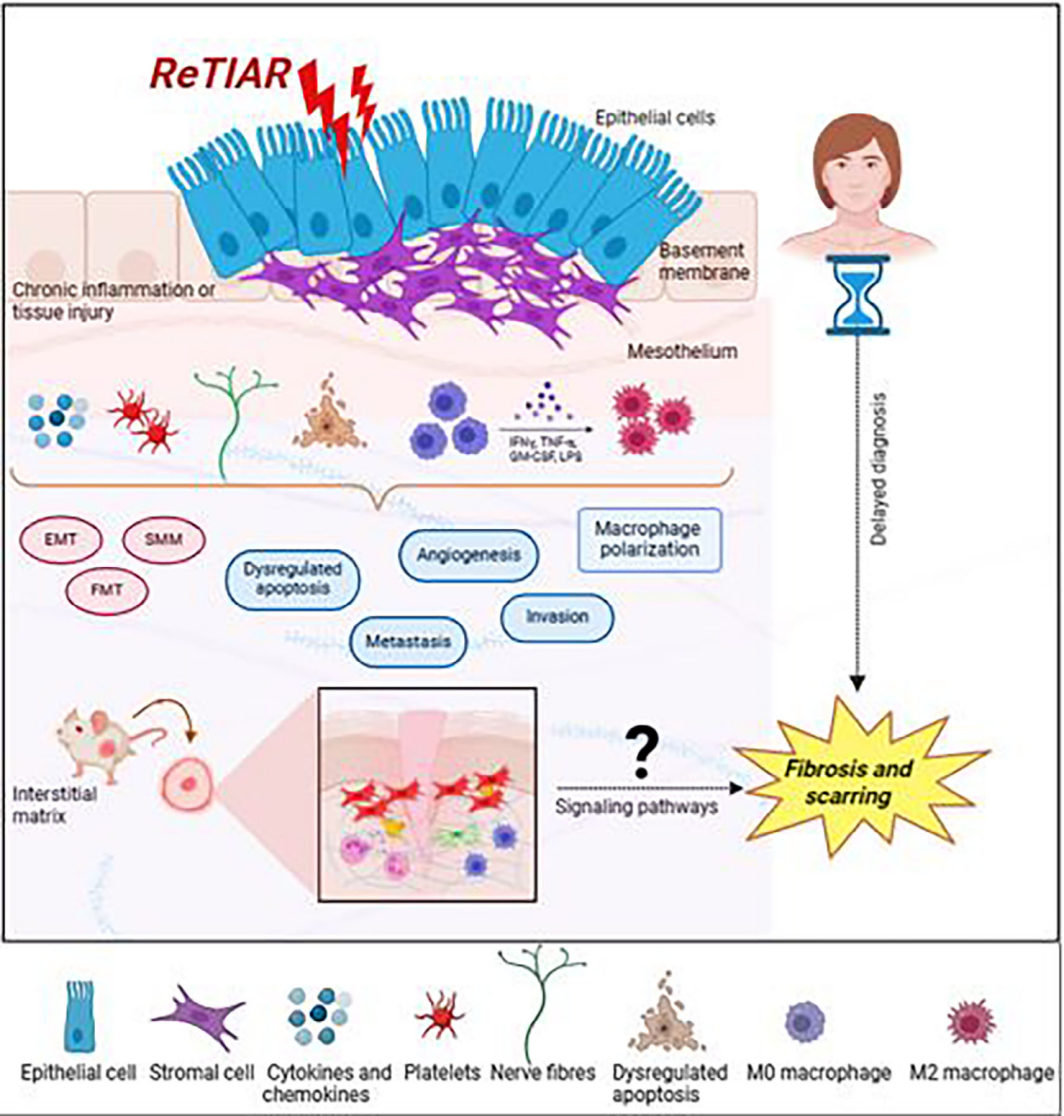
Schematic illustration of the endometriotic lesion milieu and variables expressed contributing to the progression of peritoneal endometriosis (created with Biorender.com).

Mice are the most popular experimental animal models because of their ease of gene manipulation, availability, and handling, tissue similarity *in vivo*, small size and large litter, which make them cost-effective, and their relatively short gestation, which allows transgenerational examination. ^52^ On the basis of the vast majority of already available research publications, two types of mouse models have been successfully used to implant endometriotic lesions. The first approach involves suturing, whereby human endometriotic implants are surgically autotransplanted into the peritoneum of immunocompromised mice. ^77– 79^ The second approach involves the intraperitoneal or subcutaneous implantation of autologous uterine segments into the peritoneum of recipient mice from a syngeneic donor. ^47^^,^^80^^,^^81^ Although there are numerous reports describing the spontaneous attachment, growth, and proliferation of endometriotic lesions, these lesions do not accurately reflect human endometriosis because they do not exhibit characteristics such as chronic, persistent fibrosis for internal scarring, or invasiveness resulting from EMT. Moreover, these animal models provide data on the inflammatory processes generated by implanted lesions rather than those caused by endometriosis. Rats can only produce superficial lesions, which are the most fundamental and possibly least clinically significant types of lesions. The inability of any study to recreate fibrotic endometriotic lesions may account for the failure of rat models to yield data relevant to the pathophysiology and treatment of human endometriosis. This situation demonstrates that the preclinical animal studies that have been established are not transferable. ^82^ Many studies using rodents as a model for endometriosis have investigated the gene expression patterns of ectopic tissue deposits in rats in an attempt to correlate them with human endometriotic lesions. Chronic inflammation, angiogenesis, and extracellular matrix remodeling are common pathways. ^83– 85^ While some aspects of the disease are replicated in the rodent model, all the modifications involve suturing uterine fragments (endometrium plus myometrium) to different sites, which does not accurately represent the formation of lesions from those shed endometrial tissue or the dissemination of menstrual tissue into the peritoneum. Notably, particularly in terms of understanding its pathophysiology and treatment options, the current rodent models have not been successful in yielding findings that apply to human endometriosis. Therefore, fibrosis, a mostly disregarded component of human endometriosis, should be taken into consideration. ^86^^,^^87^ However, efforts to translate the results into humans were unsuccessful in offering effective endometriosis treatments. Therefore, developing novel rodent models that mirror the continuous fibrotic process observed in endometriotic patients is essential for improving our understanding of this disease. An increasing amount of research has recently focused on the role that fibrosis plays in clinical-grade endometriosis. On the other hand, little is known about fibrosis treatment strategies. Therefore, developing a fibrotic mouse model of endometriosis, elucidating the regulatory processes underlying fibrosis in endometriosis, and identifying more precise specific biomarkers for this disease are critical. These markers can also be utilized to find effective therapeutic targets and identify endometriosis in its early phases ( Figure 2).

To mimic the fibrotic scarring observed in endometriosis, many endometriotic fibrotic animal models have been developed ( Table 1). Furthermore, new *in vivo* models that use stromal cells generated from menstrual blood have been created to study endometriosis; these models show enhanced endometriotic cell migration and proliferation. ^58^ Many cues, including estrogen stimulation, may trigger EMT. ^88^ Furthermore, estrogen-induced EMT in Ishikawa cells promotes adenomyosis. ^89^ However, how estrogen causes EMT in endometriosis at the molecular level remains unknown. To prevent fluctuations in mouse estradiol levels during the estrous cycle, the majority of established mouse models use ovariectomized mice. ^90– 93^ As a result, the steady availability of estradiol in the circulation may help promote lesion establishment and growth. However, research on how estrogen-induced EMT in endometriosis affects fertility, such as in women with normal circulating estrogen, is impossible. Therefore, studies of endometriosis produced in intact mice call for more research on the connection between ectopic tissue and fertility.

**Table 1.** This paper outlines rodent research on endometriosis and the genes associated with fibrosis.

Model	Fibrotic gene	Mechanism	References
BALB/c	TGF-β1, p-Smad3, CCN2, α-SMA, collagen I and LOX	EMT, FMT, SMM and fibrosis	^90^
BALB/c	PCNA, VEGF, collagen I, a-SMA, and LOX	FMT and fibrosis	^94^
BALB/c	β-catenin	Fibrosis	^95^
BALB/c	VEGF, MVD, PCNA, p-p65, COX-2, TGF-β1, α-SMA, and collagen I	Fibrosis	^92^
C57BL/6	VEGF, PCNA, COX-2, p-p65, collagen I, α-SMA, Fibronectin, FGFR2, MVD, Platelets	Inflammation and fibrosis	^91^
C57BL/6	TGF-β and KLF 10 and 11	Fibrosis	^96^^,^^97^
C57/BL6	COL1A1/Col1a1	Fibrosis	^98^
C57BL/6	Nrf2	Fibrogenesis	^99^
C57BL/6	PIM2	Fibrosis	^100^

### Human experiment details

After years of relentless advocacy from individuals affected by the condition, endometriosis is gradually gaining increased attention, as evidenced by an increase in research, particularly large-scale controlled human trials and meta-analyses, which have the potential to significantly increase awareness of the condition and its management. In endometriotic lesions, TGF-β family members, the Notch receptor, and the bioactive sphingolipid sphingosine 1-phosphate (S1P) cause tissue fibrosis and change signaling pathways. ^96^ NF-κB is activated in endometriotic lesions and peritoneal macrophages, which are essential for the inflammation associated with endometriosis. It has been demonstrated that inhibiting NF-κB decreases the development and progression of endometriosis in women as well as its associated symptoms. ^101^ Estrogen can promote the formation and dissemination of endometriosis ectopic lesions by upregulating the expression of the transcription factor Slug in ectopic endothelial cells and inducing EMT. ^89^ Fibrogenesis in endometriosis may be facilitated by aberrant Wnt/β-catenin pathway activation and reversed by blocking the Wnt/β-catenin pathway. ^98^ TGF-β1 may stimulate the expression of N-cadherin, OCT4, and Snail in ectopic stromal cells, suggesting that TGF-β1 facilitates cell invasion. ^99^ The AKT and ERK signaling pathways may work synergistically to promote the formation of deep endometriotic lesions by increasing endometriotic stromal cell proliferation in a fibrotic milieu *in vitro*. ^94^

By inducing EMT and FMT in endometriotic lesions, platelet-derived TGF-β1 stimulates SMM and fibrosis. ^95^ Evidence suggests that EMT induces fibrogenesis in addition to increasing cellular invasiveness. For example, the TGF-β1/Smad3 signaling pathway, which is driven by platelets, is known to induce EMT and FMT in endometriotic lesions, which eventually results in SMM and fibrosis. ^95^ Targeting TGF-β1 may be an effective strategy to prevent fibrosis and adhesion formation since endometriotic cells release TGF-β1, which induces ECM disorganization and fibrosis in the tissues of ovarian endometriotic patients. ^100^ Oxidative stress has been linked to the ADAM17/Notch signaling pathway and perhaps fibrosis, according to a study performed on endometriosis patients. ^97^ Furthermore, endometriotic cells of endometriomas express Smad2, Smad3, and Smad4 (as well as their phosphorylated forms), which causes fibrosis and adhesion to ovarian tissues, suggesting a role for TGF-β1/Smad signaling. ^100^ FOXP1 uses Wnt signaling to increase fibrosis in endometriosis. ^102^ Through their effects on tissue repair, senescence, EMT, FMT, and proliferation of fibroblasts/myofibroblasts, mutations in TP53, PTEN, ARID1A, PIK3CA, KRAS, and PPP2R1A promote the development and fibrogenesis of endometriosis. ^103^ A significant increase in the mRNA levels of α-SMA, vimentin, N-cadherin, fibronectin, PAI-1 (Serpine1), Snail, Slug, and LOX was detected. ^104^ Growth factors such as TGF-β1, PDGF, EGF, and CTGF are released by activated platelets in lesions, facilitating fibrogenesis in endometriotic patients with deep endometriosis and ovarian endometrioma. ^105^ NR4A1 is a novel pro-endometriotic transcription factor that accelerates the development of endometriosis. ^106^ HOXC8 stimulates TGF-β signaling, which affects adhesion, cell proliferation, migration, and ovarian endometrioma. ^107^ FAK, an intracellular nonreceptor tyrosine kinase, mediates a series of processes involved in the development of endometriosis, including cell adhesion, the inflammatory response, and fibrosis signaling, in patients with endometriomas. ^108^ In ectopic ESCs derived from retrograde menstruation, PGE2/thrombin is known to induce modifications such as FMT and EMT, which are linked to fibrotic changes in lesions. Through the EMT and FMT processes, proinflammatory substances such as PGE2 and thrombin in retrograde menstrual fluid are jointly implicated in generating endometriosis fibrosis in endometriotic patients. These findings suggest potential targets for treatment to mitigate fibrosis. ^36^ In addition to fibrotic and EMT markers, numerous processes, such as pyroptosis, the NLRP3 inflammasome, and deregulation of the long noncoding RNA MALAT1, have been shown to cause fibrosis in endometriotic patients. ^90^ Reducing the number of lesions by targeting inflammatory molecules such as IL-8 is also known to reduce fibrosis and adhesions, highlighting the potential for disease-modifying therapy. ^109^ Although existing studies have shed light on identifying genes associated with fibrosis in endometriosis, further exploration of the intricate signaling networks underlying this condition remains necessary. This gap underscores the necessity for future research employing advanced methodologies such as knockout models, high-throughput RNA sequencing, and omics techniques. These techniques provide clearer insights into the mechanisms of fibrotic indicators and assist in validating their function in endometriosis development, providing solid evidence for the discovery of drugs that hinder, terminate, and reverse fibrosis progression and benefit endometriotic patients.

### Interplay of EMT and MMPs in endometriosis

Endometriosis is a common benign gynecological disease with a high propensity for migration and invasion. The cell-to-cell or cell-ECM connections allow the cells to migrate, invade, and proliferate in new locations. MMPs are linked to adhesion, invasion, and the severity of endometriosis. These findings indicate that MMPs play a role in extracellular matrix remodeling, which is necessary for the development of ectopic endometriosis lesions. ^110^ They are also significantly more abundant in the endometrial and peritoneal fluid of endometriosis patients. ^111^^,^^112^ Matrix metalloproteinases (MMPs) are a family of enzymes that are mostly found in the functional layer of the endometrium. They are secreted by resident immune cells and stromal fibroblasts, which facilitate the remodeling of the extracellular matrix, including collagen, elastins, and other glycoproteins, and endometrial disintegration during menstruation. Tissue inhibitors of matrix metalloproteinases (TIMPs) are endogenous antagonists that reduce MMP overexpression, and ovarian steroid hormones are known to control MMP activity. ^113^ For early clinical studies of EMT, the nude mouse is a suitable model, particularly for the identification of MMP-2 and TIMP-2, proteins that seem to play a significant role in the pathophysiology of EMT. Estrogen specifically increases MMP-2 expression to encourage ectopic implantation of the endometrium. On the other hand, progestin can suppress TIMP-2 expression, increasing the MMP-2/TIMP-2 ratio and increasing the invasiveness of the ectopic endometrium to facilitate implantation. ^114^ In ovarian endometriosis, MMP7 facilitates EMT; EGF increases MMP7 expression by activating the ERK1–AP1 pathway ^115^^,^^116^ MMP14 affects the development and function of invadopodia, which in turn modulates the ability of mesenchymal cells to invade and migrate. ^117^ MMP-2 and MMP-9, two important enzymes involved in the destruction of diverse types of ECM, have been linked to the development of endometriosis by regulating endometrial cell invasion. ^118^ Both MMP-2 and MMP-9 have been shown to function as biomarkers of both EMT and triggering factors that contribute to the progression of EMT. ^119^ As a result, we hypothesize that MMPs may be crucial in controlling the endometriosis-related EMT process. However, further research is needed to fully understand the connection between MMPs and EMT in fibrotic endometriosis, as there are not enough comprehensive studies on this topic. Despite this, it is apparent that MMPs play crucial roles in collagen production, which is necessary for the gradual development of endometriosis fibrosis. ^106^ These findings suggest that there may be a precise equilibrium between collagen synthesis and breakdown, which should be investigated further. As a result, we hypothesize that MMPs may be crucial in controlling the endometriosis-related EMT process. However, further research is needed to fully understand the connection between MMPs and EMT-induced fibrosis in endometriosis, as there are not enough comprehensive studies on this topic.

## Discussion

Endometriosis is an underdiagnosed chronic inflammatory disease that affects millions of people around the world. The primary explanation for endometriosis growth is the transplantation of living endometrial cells that are refluxed after menstruation, thereby attaching to and invading other pelvic organs and leading to inflammation and fibrosis. ^2^ Despite its broad incidence and importance, endometriosis research has significant limitations. ^120^ The gaps include a lack of understanding of the disease’s etiology, a delay in diagnosis that necessitates invasive treatments, and the difficulties of integrating electronic health records for research, which aids in identifying potential therapeutic tools and reminds us to look beyond endometriotic lesions. ^121^ Currently, 50 to 70% of endometriotic drugs that have advanced to phases II and III in clinical trials are unable to show efficacy, suggesting an unfulfilled research gap in the development of appropriate animal models. ^119^ Endometrotic fibrosis shares characteristics with other fibrotic conditions, including increased myofibroblast and smooth muscle cell activity, high levels of fibrotic-associated growth factor and protein production, epithelial-to-mesenchymal transition, and collagen deposition. ^16^ There is substantial evidence that fibrosis is a molecular characteristic of endometriosis etiology along with other molecular hallmarks, such as immunological dysregulation, ER expression, progesterone resistance, chronic inflammation, angiogenesis, and epigenetic changes. ^16^ Interestingly, fibrosis, as a histologic feature of lesions, can progress, most likely due to repeated tissue injury and repair caused by inflammation-induced recurrent menstrual bleeding. ^25^^,^^120^ Thus, a thorough understanding of the disease process is needed for progress in the fields of biomarker identification and nonhormonal therapy. Fibrosis may impair drug administration and efficacy. Rather, a study into the mechanisms that resolve fibrosis will uncover new possibilities by discovering new targets for pharmacologically regulating this condition, notably in the pharmacology of multicomponent medications. ^103^^,^^121^ Because EMT-induced fibrosis is numerous and diverse and plays vital functions in various human body systems, robust longitudinal studies are needed to [a] confirm biomarkers and underlying mechanisms linked with fibrosis progression, providing insights into disease causes and potential diagnostic or prognostic tools. [b] To investigate temporal dynamics to record the advancement of fibrosis over time, researchers can better comprehend its development from early stages to advanced stages, thereby allowing early intervention and personalized treatment methods. [c] Investigating treatment efficacy, or the effectiveness of various interventions for fibrosis, can provide useful data on long-term outcomes and responses. [d] To better understand the natural course of fibrosis, including its variations among individuals, potential triggers, and variables influencing its progression, preventive and targeted therapeutics should be created. [e] To determine whether the inflammatory environment of endometriosis is involved in fibrosis. The proposed pathways by which endometriosis participates in fibrosis require further investigation. Indeed, discovering fibrosis-specific therapies for endometriosis remains a significant issue. As a result, further inquiry and investigation are needed in the future. Identifying the underlying etiology of endometriosis is more difficult because the disease’s missing components, such as EMT-induced fibrosis, have yet to be replicated in experimental rodent models. Filling these gaps may lead to more accurate patient diagnoses, more effective medications, and better knowledge of how the disorder affects women’s lives. Any treatments that help reduce the fibrotic aspect of the disease will have far-reaching implications for the individual, the population, and the healthcare system. These thought-provoking articles show our reliance on carefully selected animal models to advance our understanding of endometriosis. These findings emphasize the multisystem characteristics of endometriosis, as well as the need for researchers to think beyond only the endometrial lesion. As we have come to expect, no single cause can explain endometriosis, yet these studies suggest that more therapeutic methods to improve the quality of life for affected people are needed. This breakthrough in the construction of models represents promising research that could have substantial beneficial consequences for patients. Translating these research findings into clinical care will undoubtedly aid in shortening the extended delay to diagnosis and understanding the epidemiological underpinnings of the condition.

## Conclusion

Endometriosis is a prevalent gynecological condition that significantly affects the physical and emotional well-being of female patients because of its invasive and recurrent characteristics. Fibrosis, as a histological characteristic of lesions, may progress, presumably due to recurrent tissue injury and repair. In a nonhuman primate model of endometriosis, the predominant type of peritoneal lesion transitioned from red vesicular to white fibrotic over the course of time. However, the association between endometriosis and fibrosis is poorly understood. Additionally, EMT may play a role in the etiology of endometriosis through immunological regulation, the production of proinflammatory cytokines, and other mechanisms. Clinical trials have shown that targeting EMT-induced fibrosis can help treat endometriosis, establishing a new research direction and theoretical foundation for the diagnosis and treatment of fibrotic endometriotic patients. As randomized, double-blinded investigations of endometriosis in women are difficult and at times ethically restrictive, animal models for endometriosis have evolved into vital tools for obtaining a mechanical understanding of the etiology and pathophysiology mechanisms of this complex condition. Thus, it is vital to examine the molecular pathways that drive and sustain fibrosis in endometriosis via a novel fibrosis-based animal model to discover new pharmacological targets and provide creative therapeutics for patients. Furthermore, the research connecting endometriosis and fibrosis has added a further complicating factor to the shared strategy for dealing with endometriotic patients with infertility, as well as a potentially essential concern in the counseling and management of the condition for those desiring future fertility. Well-designed longitudinal studies are needed to improve clinical decision-making in these contexts. Although gynecological surgeons are aware of the complex role of fibrosis in the surgical treatment of endometriosis, the molecular pathways that relate fibrosis to endometriosis-associated pain and infertility remain unknown. Thus, more research is needed to better understand the clinical implications of fibrosis and identify it as a molecular marker of endometriosis etiology, a potentially important element to consider when counseling and managing endometriotic patients who are planning to have children in the future. Well-designed longitudinal studies are needed to make more informed clinical decisions in these contexts. Efforts should be focused on building trustworthy models that incorporate physiologically relevant cells, such as organoids and microfluidics. The continued creation of mouse models to aid in understanding the processes of endometriosis development offers the best chance of creating therapeutic options to prevent or reverse this mysterious disease. This review aims to spark a debate on the need to revise the present understanding by focusing on the fibrotic features of endometriosis pathogenesis. We believe that this approach will shed new light on this condition and suggest areas that need to be investigated further.

## Data Availability

No data are associated with this article.

## References

[1] Bonavina G Taylor HS : Endometriosis-associated infertility: From pathophysiology to tailored treatment. *Front Endocrinol.* 2022;13:1020827. 10.3389/fendo.2022.1020827 36387918 PMC9643365

[2] Sourial S Tempest N Hapangama DK : Theories on the pathogenesis of endometriosis. *Int J Reprod Med.* 2014;2014:1–9. 10.1155/2014/179515 PMC4334056 25763392

[3] Gajbhiye RK Montgomery G Pai MV : Protocol for a case-control study investigating the clinical phenotypes and genetic regulation of endometriosis in Indian women: the ECGRI study. *BMJ Open.* 2021 Aug 9;11(8):e050844. 10.1136/bmjopen-2021-050844 34373312 PMC8354274

[4] Yen CF Kim MR Lee CL : Epidemiologic Factors Associated with Endometriosis in East Asia. *Gynecol Minim Invasive Ther.* 2019;8(1):4–11. 10.4103/GMIT.GMIT_83_18 30783582 PMC6367920

[5] Alson S Henic E Jokubkiene L : Endometriosis diagnosed by ultrasound is associated with lower live birth rates in women undergoing their first in vitro fertilization/intracytoplasmic sperm injection treatment. *Fertil Steril.* 2024 May 1;121(5):832–841. 10.1016/j.fertnstert.2024.01.023 38246403

[6] Taylor HS : Endometriosis: a complex systemic disease with multiple manifestations. *Fertil Steril.* 2019 Aug;112(2):235–236. 10.1016/j.fertnstert.2019.06.006 31280952

[7] Gajbhiye RK : Endometriosis and inflammatory immune responses: Indian experience. *Am J Reprod Immunol N Y N 1989.* 2023 Feb;89(2):e13590. 10.1111/aji.13590 PMC7615030 35751585

[8] Sampson JA : Metastatic or Embolic Endometriosis, due to the Menstrual Dissemination of Endometrial Tissue into the Venous Circulation. *Am J Pathol.* 1927 Mar;3(2):93–110.43. 19969738 PMC1931779

[9] Taylor HS Kotlyar AM Flores VA : Endometriosis is a chronic systemic disease: clinical challenges and novel innovations. *Lancet Lond Engl.* 2021 Feb 27;397(10276):839–852. 10.1016/S0140-6736(21)00389-5 33640070

[10] Horne AW Missmer SA : Pathophysiology, diagnosis, and management of endometriosis. *BMJ.* 2022 Nov 14;379:e070750. 10.1136/bmj-2022-070750 36375827

[11] Malvezzi H Marengo EB Podgaec S : Endometriosis: current challenges in modeling a multifactorial disease of unknown etiology. *J Transl Med.* 2020 Aug 12;18(1):311. 10.1186/s12967-020-02471-0 32787880 PMC7425005

[12] Bafort C Beebeejaun Y Tomassetti C : Laparoscopic surgery for endometriosis. *Cochrane Database Syst Rev.* 2020 Oct 23;10(10):CD011031. 10.1002/14651858.CD011031.pub3 33095458 PMC8428328

[13] Canis M Donnez JG Guzick DS : Revised American Society for Reproductive Medicine classification of endometriosis: 1996. *Fertil Steril.* 1997 May;67(5):817–821. 10.1016/S0015-0282(97)81391-X 9130884

[14] Somigliana E Vigano P Benaglia L : Adhesion prevention in endometriosis: a neglected critical challenge. *J Minim Invasive Gynecol.* 2012;19(4):415–421. 10.1016/j.jmig.2012.03.004 22575862

[15] Vigano P Candiani M Monno A : Time to redefine endometriosis including its pro-fibrotic nature. *Hum Reprod Oxf Engl.* 2018 Mar 1;33(3):347–352. 10.1093/humrep/dex354 29206943

[16] Yang H Kang K Cheng C : Integrative Analysis Reveals Regulatory Programs in Endometriosis. *Reprod Sci.* 2015 Sep 1;22(9):1060–1072. 10.1177/1933719115592709 26134036 PMC5933170

[17] Ahn SH Monsanto SP Miller C : Pathophysiology and Immune Dysfunction in Endometriosis. *Biomed Res Int.* 2015;2015:795976. 26247027 10.1155/2015/795976 PMC4515278

[18] Herington JL Bruner-Tran KL Lucas JA : Immune interactions in endometriosis. *Expert Rev Clin Immunol.* 2011 Sep;7(5):611–626. 10.1586/eci.11.53 21895474 PMC3204940

[19] Czyzyk A Podfigurna A Szeliga A : Update on endometriosis pathogenesis. *Minerva Ginecol.* 2017 Oct;69(5):447–461. 10.23736/S0026-4784.17.04048-5 28271702

[20] Balasubramanian V Saravanan R Joseph LD : Molecular dysregulations underlying the pathogenesis of endometriosis. *Cell Signal.* 2021 Dec;88:110139. 10.1016/j.cellsig.2021.110139 34464692

[21] Laudanski P Szamatowicz J Ramel P : Matrix metalloproteinase-13 and membrane type-1 matrix metalloproteinase in peritoneal fluid of women with endometriosis. *Gynecol Endocrinol Off J Int Soc Gynecol Endocrinol.* 2005 Aug;21(2):106–110. 10.1080/09513590500154043 16109597

[22] Laudanski P Charkiewicz R Kuzmicki M : Profiling of selected angiogenesis-related genes in proliferative eutopic endometrium of women with endometriosis. *Eur J Obstet Gynecol Reprod Biol.* 2014 Jan;172:85–92. 10.1016/j.ejogrb.2013.10.007 24188612

[23] Garcia Garcia JM Vannuzzi V Donati C : Endometriosis: Cellular and Molecular Mechanisms Leading to Fibrosis. *Reprod Sci Thousand Oaks Calif.* 2023 May;30(5):1453–1461. 10.1007/s43032-022-01083-x 36289173 PMC10160154

[24] Koninckx PR Fernandes R Ussia A : Pathogenesis Based Diagnosis and Treatment of Endometriosis. *Front Endocrinol.* 2021;12:745548. 10.3389/fendo.2021.745548 34899597 PMC8656967

[25] Molina M Moreno GA Singh R : Rectovaginal endometriosis with nodular smooth muscle metaplasia diagnosed via transrectal ultrasound-guided fine-needle aspiration cytology: An underused minimally invasive diagnostic technique? *Diagn Cytopathol.* 2023 Oct;51(10):E273–E278. 10.1002/dc.25183 37318678

[26] Muzii L Bianchi A Bellati F : Histologic analysis of endometriomas: what the surgeon needs to know. *Fertil Steril.* 2007 Feb;87(2):362–366. 10.1016/j.fertnstert.2006.06.055 17094980

[27] Meng XM Nikolic-Paterson DJ Lan HY : TGF-β: the master regulator of fibrosis. *Nat Rev Nephrol.* 2016 Jun;12(6):325–338. 10.1038/nrneph.2016.48 27108839

[28] Soni UK Chadchan SB Kumar V : A high level of TGF-B1 promotes endometriosis development via cell migration, adhesiveness, colonization, and invasiveness. *Biol Reprod.* 2019 Apr 1;100(4):917–938. 10.1093/biolre/ioy242 30423016

[29] Tarokh M Ghaffari Novin M Poordast T : Serum and Peritoneal Fluid Cytokine Profiles in Infertile Women with Endometriosis. *Iran J Immunol IJI.* 2019 Jun;16(2):151–162. 10.22034/IJI.2019.80258 31182689

[30] Scutiero G Iannone P Bernardi G : Oxidative Stress and Endometriosis: A Systematic Review of the Literature. *Oxidative Med Cell Longev.* 2017;2017:7265238. 10.1155/2017/7265238 PMC5625949 29057034

[31] Zhang Q Duan J Olson M : Cellular Changes Consistent With Epithelial-Mesenchymal Transition and Fibroblast-to-Myofibroblast Transdifferentiation in the Progression of Experimental Endometriosis in Baboons. *Reprod Sci Thousand Oaks Calif.* 2016 Oct;23(10):1409–1421. 10.1177/1933719116641763 27076446 PMC5933178

[32] De Graaff AA Dirksen CD Simoens S : Quality of life outcomes in women with endometriosis are highly influenced by recruitment strategies. *Hum Reprod Oxf Engl.* 2015 Jun;30(6):1331–1341. 10.1093/humrep/dev084 25908657

[33] Asante A Taylor RN : Endometriosis: the role of neuroangiogenesis. *Annu Rev Physiol.* 2011;73:163–182. 10.1146/annurev-physiol-012110-142158 21054165

[34] Henderson NC Rieder F Wynn TA : Fibrosis: from mechanisms to medicines. *Nature.* 2020 Nov;587(7835):555–566. 10.1038/s41586-020-2938-9 33239795 PMC8034822

[35] Thiery JP Acloque H Huang RYJ : Epithelial-mesenchymal transitions in development and disease. *Cell.* 2009 Nov 25;139(5):871–890. 10.1016/j.cell.2009.11.007 19945376

[36] Itoga T Matsumoto T Takeuchi H : Fibrosis and smooth muscle metaplasia in rectovaginal endometriosis. *Pathol Int.* 2003 Jun;53(6):371–375. 10.1046/j.1440-1827.2003.01483.x 12787311

[37] Laschke MW Menger MD : Basic mechanisms of vascularization in endometriosis and their clinical implications. *Hum Reprod Update.* 2018 Mar 1;24(2):207–224. 10.1093/humupd/dmy001 29377994

[38] Zhang Q Duan J Liu X : Platelets drive smooth muscle metaplasia and fibrogenesis in endometriosis through epithelial-mesenchymal transition and fibroblast-to-myofibroblast transdifferentiation. *Mol Cell Endocrinol.* 2016 Jun 15;428:1–16. 10.1016/j.mce.2016.03.015 26992563

[39] Yang J Weinberg RA : Epithelial-mesenchymal transition: at the crossroads of development and tumor metastasis. *Dev Cell.* 2008 Jun;14(6):818–829. 10.1016/j.devcel.2008.05.009 18539112

[40] Owusu-Akyaw A Krishnamoorthy K Goldsmith LT : The role of mesenchymal-epithelial transition in endometrial function. *Hum Reprod Update.* 2019 Jan 1;25(1):114–133. 10.1093/humupd/dmy035 30407544

[41] Kusama K Fukushima Y Yoshida K : PGE2 and Thrombin Induce Myofibroblast Transdifferentiation via Activin A and CTGF in Endometrial Stromal Cells. *Endocrinology.* 2021 Dec 1;162(12):bqab207. 10.1210/endocr/bqab207 34606582

[42] Proestling K Birner P Gamperl S : Enhanced epithelial to mesenchymal transition (EMT) and upregulated MYC in ectopic lesions contribute independently to endometriosis. *Reprod Biol Endocrinol RBE.* 2015 Jul 22;13:75. 10.1186/s12958-015-0063-7 26198055 PMC4511248

[43] Mishra A Galvankar M Vaidya S : Mouse model for endometriosis is characterized by proliferation and inflammation but not epithelial-to-mesenchymal transition and fibrosis. *J Biosci.* 2020;45:105. 10.1007/s12038-020-00073-y 32975232

[44] Wibisono H Nakamura K Taniguchi F : Tracing location by applying Emerald luciferase in an early phase of murine endometriotic lesion formation. *Exp Anim.* 2022 May 20;71(2):184–192. 10.1538/expanim.21-0146 34819403 PMC9130045

[45] García-Gómez E Vázquez-Martínez ER Reyes-Mayoral C : Regulation of Inflammation Pathways and Inflammasome by Sex Steroid Hormones in Endometriosis. *Front Endocrinol.* 2019;10:935. 10.3389/fendo.2019.00935 PMC7000463 32063886

[46] Miller JE Monsanto SP Ahn SH : Interleukin-33 modulates inflammation in endometriosis. *Sci Rep.* 2017 Dec 20;7(1):17903. 10.1038/s41598-017-18224-x 29263351 PMC5738435

[47] Giacomini E Minetto S Li Piani L : Genetics and Inflammation in Endometriosis: Improving Knowledge for Development of New Pharmacological Strategies. *Int J Mol Sci.* 2021 Aug 21;22(16):9033. 10.3390/ijms22169033 34445738 PMC8396487

[48] Cordaro M Trovato Salinaro A Siracusa R : Hidrox ^®^ and Endometriosis: Biochemical Evaluation of Oxidative Stress and Pain. *Antioxidants.* 2021 May 4;10(5):720. 10.3390/antiox10050720 34064310 PMC8147870

[49] Lu H Hu H Yang Y : The inhibition of reactive oxygen species (ROS) by antioxidants inhibits the release of an autophagy marker in ectopic endometrial cells. *Taiwan J Obstet Gynecol.* 2020 Mar;59(2):256–261. 10.1016/j.tjog.2020.01.014 32127147

[50] Greaves E Cousins FL Murray A : A novel mouse model of endometriosis mimics human phenotype and reveals insights into the inflammatory contribution of shed endometrium. *Am J Pathol.* 2014 Jul;184(7):1930–1939. 10.1016/j.ajpath.2014.03.011 24910298 PMC4076466

[51] Tanbo T Fedorcsak P : Endometriosis-associated infertility: aspects of pathophysiological mechanisms and treatment options. *Acta Obstet Gynecol Scand.* 2017 Jun;96(6):659–667. 10.1111/aogs.13082 27998009

[52] Perrone U Evangelisti G Laganà AS : A review of phase II and III drugs for the treatment and management of endometriosis. *Expert Opin Emerg Drugs.* 2023 Dec;28(4):333–351. 10.1080/14728214.2023.2296080 38099328

[53] Fan H : *In-vitro* models of human endometriosis. *Exp Ther Med.* 2020 Mar;19(3):1617–1625. 10.3892/etm.2019.8363 32104212 PMC7027135

[54] Bruner-Tran KL Eisenberg E Yeaman GR : Steroid and cytokine regulation of matrix metalloproteinase expression in endometriosis and the establishment of experimental endometriosis in nude mice. *J Clin Endocrinol Metab.* 2002 Oct;87(10):4782–4791. 10.1210/jc.2002-020418 12364474

[55] D’Hooghe TM Debrock S Hill JA : Endometriosis and subfertility: is the relationship resolved? *Semin Reprod Med.* 2003 May;21(2):243–254. 10.1055/s-2003-41330 12917793

[56] Chiorean DM Mitranovici MI Toru HS : New Insights into Genetics of Endometriosis-A Comprehensive Literature Review. *Diagn Basel Switz.* 2023 Jul 4;13(13):2265. 10.3390/diagnostics13132265 PMC10340419 37443659

[57] Abramiuk M Grywalska E Małkowska P : The Role of the Immune System in the Development of Endometriosis. *Cells.* 2022 Jun 25;11(13):2028. 10.3390/cells11132028 35805112 PMC9265783

[58] Zhang Y Ma NY : Environmental Risk Factors for Endometriosis: An Umbrella Review of a Meta-Analysis of 354 Observational Studies With Over 5 Million Populations. *Front Med.* 2021;8:680833. 10.3389/fmed.2021.680833 34760897 PMC8573094

[59] Coiplet E Courbiere B Agostini A : Endometriosis and environmental factors: A critical review. *J Gynecol Obstet Hum Reprod.* 2022 Sep;51(7):102418. 10.1016/j.jogoh.2022.102418 35667590

[60] Zhang Y He T Lin T : Novel in vivo endometriotic models associated eutopic endometrium by implanting menstrual blood-derived stromal cells from patients with endometriosis. *Sci Rep.* 2023 May 23;13(1):8347. 10.1038/s41598-023-35373-4 37221282 PMC10206158

[61] Mori T Ito F Koshiba A : Local estrogen formation and its regulation in endometriosis. *Reprod Med Biol.* 2019 Oct;18(4):305–311. 10.1002/rmb2.12285 31607790 PMC6780031

[62] Becker CM Gattrell WT Gude K : Reevaluating response and failure of medical treatment of endometriosis: a systematic review. *Fertil Steril.* 2017 Jul;108(1):125–136. 10.1016/j.fertnstert.2017.05.004 28668150 PMC5494290

[63] Fazleabas A : Models of Endometriosis: Animal Models II - Non-Human Primates. *Endometriosis: Science and Practice.* 2012; pp.285–291. 10.1002/9781444398519.ch27

[64] Hastings JM Fazleabas AT : A baboon model for endometriosis: implications for fertility. *Reprod Biol Endocrinol RBE.* 2006;4(Suppl 1)):S7. 10.1186/1477-7827-4-S1-S7 PMC1775067 17118171

[65] Kyama CM Mihalyi A Chai D : Baboon model for the study of endometriosis. *Womens Health Lond Engl.* 2007 Sep;3(5):637–646. 10.2217/17455057.3.5.637 19804041

[66] Dehoux JP Defrère S Squifflet J : Is the baboon model appropriate for endometriosis studies? *Fertil Steril.* 2011 Sep;96(3):728–733.e3. 10.1016/j.fertnstert.2011.06.037 21774926

[67] Zondervan K Cardon L Desrosiers R : The genetic epidemiology of spontaneous endometriosis in the rhesus monkey. *Ann N Y Acad Sci.* 2002 Mar;955:233–238. discussion 293-295, 396–406. 10.1111/j.1749-6632.2002.tb02784.x 11949951

[68] Wilson RC Link JM Lee YZ : Uterine Uptake of Estrogen and Progestogen-Based Radiotracers in Rhesus Macaques with Endometriosis. *Mol Imaging Biol.* 2024 Apr;26(2):334–343. 10.1007/s11307-023-01892-9 38133866 PMC11034810

[69] Nishimoto-Kakiuchi A Netsu S Matsuo S : Characteristics of histologically confirmed endometriosis in cynomolgus monkeys. *Hum Reprod Oxf Engl.* 2016 Oct;31(10):2352–2359. 10.1093/humrep/dew209 27591226 PMC5027930

[70] Hayashi K Nakayama M Iwatani C : The Natural History of Spontaneously Occurred Endometriosis in Cynomolgus Monkeys by Monthly Follow-Up Laparoscopy for Two Years. *Tohoku J Exp Med.* 2020 Aug;251(4):241–253. 10.1620/tjem.251.241 32713879

[71] Grimm D : US labs using a record number of monkeys. *Science.* 2018 Nov 9;362(6415):630. 10.1126/science.362.6415.630 30409868

[72] Pullen N Birch CL Douglas GJ : The translational challenge in the development of new and effective therapies for endometriosis: a review of confidence from published preclinical efficacy studies. *Hum Reprod Update.* 2011;17(6):791–802. 10.1093/humupd/dmr030 21733981

[73] Dorning A Dhami P Panir K : Bioluminescent imaging in induced mouse models of endometriosis reveals differences in four model variations. *Dis Model Mech.* 2021 Aug;14(8):dmm049070. 10.1242/dmm.049070 34382636 PMC8419713

[74] Bruner-Tran KL Mokshagundam S Herington JL : Rodent Models of Experimental Endometriosis: Identifying Mechanisms of Disease and Therapeutic Targets. *Curr Womens Health Rev.* 2018 Jun;14(2):173–188. 10.2174/1573404813666170921162041 29861705 PMC5925870

[75] Fortin M Lépine M Pagé M : An improved mouse model for endometriosis allows noninvasive assessment of lesion implantation and development. *Fertil Steril.* 2003 Sep;80 Suppl 2:832–838. 10.1016/S0015-0282(03)00986-5 14505761

[76] Lee B Du H Taylor HS : Experimental murine endometriosis induces DNA methylation and altered gene expression in eutopic endometrium. *Biol Reprod.* 2009 Jan;80(1):79–85. 10.1095/biolreprod.108.070391 18799756 PMC2804809

[77] Burns KA Rodriguez KF Hewitt SC : Role of estrogen receptor signaling required for endometriosis-like lesion establishment in a mouse model. *Endocrinology.* 2012 Aug;153(8):3960–3971. 10.1210/en.2012-1294 22700766 PMC3404357

[78] Forster R Sarginson A Velichkova A : Macrophage-derived insulin-like growth factor-1 is a key neurotrophic and nerve-sensitizing factor in pain associated with endometriosis. *FASEB J Off Publ Fed Am Soc Exp Biol.* 2019 Oct;33(10):11210–11222. 10.1096/fj.201900797R PMC6766660 31291762

[79] Perrin S : Preclinical research: Make mouse studies work. *Nature.* 2014 Mar 27;507(7493):423–425. 10.1038/507423a 24678540

[80] Flores I Rivera E Ruiz LA : Molecular profiling of experimental endometriosis identified gene expression patterns in common with human disease. *Fertil Steril.* 2007 May;87(5):1180–1199. 10.1016/j.fertnstert.2006.07.1550 17478174 PMC1927082

[81] Konno R Fujiwara H Netsu S : Gene expression profiling of the rat endometriosis model. *Am J Reprod Immunol N Y N 1989.* 2007 Oct;58(4):330–343. 10.1111/j.1600-0897.2007.00507.x 17845203

[82] Umezawa M Sakata C Tanaka N : Cytokine and chemokine expression in a rat endometriosis is similar to that in human endometriosis. *Cytokine.* 2008 Aug;43(2):105–109. 10.1016/j.cyto.2008.04.016 18595729

[83] He Y Liang B Hung SW : Re-evaluation of mouse models of endometriosis for pathological and immunological research. *Front Immunol.* 2022;13:986202. 10.3389/fimmu.2022.986202 36466829 PMC9716019

[84] Greaves E Rosser M Saunders PTK : Endometriosis-Associated Pain - Do Preclinical Rodent Models Provide a Good Platform for Translation? *Adv Anat Embryol Cell Biol.* 2020;232:25–55. 10.1007/978-3-030-51856-1_3 33278006

[85] Jeon SY Hwang KA Choi KC : Effect of steroid hormones, estrogen and progesterone, on epithelial mesenchymal transition in ovarian cancer development. *J Steroid Biochem Mol Biol.* 2016 Apr;158:1–8. 10.1016/j.jsbmb.2016.02.005 26873134

[86] Chen YJ Li HY Huang CH : Oestrogen-induced epithelial-mesenchymal transition of endometrial epithelial cells contributes to the development of adenomyosis. *J Pathol.* 2010 Nov;222(3):261–270. 10.1002/path.2761 20814901

[87] Xu X Wang J Guo X : GPR30-mediated non-classic estrogen pathway in mast cells participates in endometriosis pain via the production of FGF2. *Front Immunol.* 2023;14:1106771. 10.3389/fimmu.2023.1106771 36845134 PMC9945179

[88] Park S Ham J Yang C : Melatonin inhibits endometriosis development by disrupting mitochondrial function and regulating tiRNAs. *J Pineal Res.* 2023 Jan;74(1):e12842. 10.1111/jpi.12842 36401340

[89] Zhu S Chen Q Sun J : The cGAS-STING pathway promotes endometriosis by up-regulating autophagy. *Int Immunopharmacol.* 2023 Apr;117:109644. 10.1016/j.intimp.2022.109644 36878046

[90] Kiani K Movahedin M Malekafzali H : Effect of the estrus cycle stage on the establishment of murine endometriosis lesions. *Int J Reprod Biomed.* 2018 May;16(5):305–314. 10.29252/ijrm.16.5.305 30027146 PMC6046203

[91] Lebman DA Spiegel S : Thematic Review Series: Sphingolipids. Cross-talk at the crossroads of sphingosine-1-phosphate, growth factors, and cytokine signaling. *J Lipid Res.* 2008 Jul;49(7):1388–1394. 10.1194/jlr.R800008-JLR200 18387885 PMC2431110

[92] González-Ramos R Van Langendonckt A Defrère S : Involvement of the nuclear factor-κB pathway in the pathogenesis of endometriosis. *Fertil Steril.* 2010 Nov;94(6):1985–1994. 10.1016/j.fertnstert.2010.01.013 20188363

[93] Matsuzaki S Darcha C : Involvement of the Wnt/β-catenin signaling pathway in the cellular and molecular mechanisms of fibrosis in endometriosis. *PLoS One.* 2013;8(10):e76808. 10.1371/journal.pone.0076808 24124596 PMC3790725

[94] Au HK Chang JH Wu YC : TGF-βI Regulates Cell Migration through Pluripotent Transcription Factor OCT4 in Endometriosis. *PLoS One.* 2015 Dec 16;10(12):e0145256. 10.1371/journal.pone.0145256 26675296 PMC4682958

[95] Matsuzaki S Darcha C : Co-operation between the AKT and ERK signaling pathways may support growth of deep endometriosis in a fibrotic microenvironment in vitro. *Hum Reprod Oxf Engl.* 2015 Jul;30(7):1606–1616. 10.1093/humrep/dev108 25976656

[96] Zhang Q Liu X Guo SW : Progressive development of endometriosis and its hindrance by anti-platelet treatment in mice with induced endometriosis. *Reprod Biomed Online.* 2017 Feb;34(2):124–136. 10.1016/j.rbmo.2016.11.006 27916451

[97] Shi LB Zhou F Zhu HY : Transforming growth factor beta1 from endometriomas promotes fibrosis in surrounding ovarian tissues via Smad2/3 signaling. *Biol Reprod.* 2017 Jan 1;97(6):873–882. 10.1093/biolre/iox140 29136085

[98] González-Foruria I Santulli P Chouzenoux S : Dysregulation of the ADAM17/Notch signalling pathways in endometriosis: from oxidative stress to fibrosis. *Mol Hum Reprod.* 2017 Jul 1;23(7):488–499. 10.1093/molehr/gax028 28486700

[99] Shao X Wei X : FOXP1 enhances fibrosis via activating Wnt/β-catenin signaling pathway in endometriosis. *Am J Transl Res.* 2018;10(11):3610–3618. 30662612 PMC6291715

[100] Guo S : Cancer driver mutations in endometriosis: Variations on the major theme of fibrogenesis. *Reprod Med Biol.* 2018 Aug 16;17(4):369–397. 10.1002/rmb2.12221 30377392 PMC6194252

[101] Yan D Liu X Xu H : Mesothelial Cells Participate in Endometriosis Fibrogenesis Through Platelet-Induced Mesothelial-Mesenchymal Transition. *J Clin Endocrinol Metab.* 2020 Nov 1;105(11):e4124–e4147. 10.1210/clinem/dgaa550 32813013

[102] Yan D Liu X Xu H : Platelets induce endothelial-mesenchymal transition and subsequent fibrogenesis in endometriosis. *Reprod Biomed Online.* 2020 Sep;41(3):500–517. 10.1016/j.rbmo.2020.03.020 32709523

[103] Mohankumar K Li X Sung N : Bis-Indole-Derived Nuclear Receptor 4A1 (NR4A1, Nur77) Ligands as Inhibitors of Endometriosis. *Endocrinology.* 2020 Apr 1;161(4):bqaa027. 10.1210/endocr/bqaa027 32099996 PMC7105386

[104] Mihara Y Maekawa R Sato S : An Integrated Genomic Approach Identifies HOXC8 as an Upstream Regulator in Ovarian Endometrioma. *J Clin Endocrinol Metab.* 2020 Dec 1;105(12):e4474–e4489. 10.1210/clinem/dgaa618 32877504

[105] Nagai T Ishida C Nakamura T : Focal Adhesion Kinase-Mediated Sequences, Including Cell Adhesion, Inflammatory Response, and Fibrosis, as a Therapeutic Target in Endometriosis. *Reprod Sci Thousand Oaks Calif.* 2020 Jul;27(7):1400–1410. 10.1007/s43032-019-00044-1 32329031

[106] Nishimoto-Kakiuchi A Sato I Nakano K : A long-acting anti-IL-8 antibody improves inflammation and fibrosis in endometriosis. *Sci Transl Med.* 2023 Feb 22;15(684):eabq5858. 10.1126/scitranslmed.abq5858 36812343

[107] Ke J Ye J Li M : The Role of Matrix Metalloproteinases in Endometriosis: A Potential Target. *Biomolecules.* 2021 Nov 22;11(11):1739. 10.3390/biom11111739 34827737 PMC8615881

[108] Protopapas A Markaki S Mitsis T : Immunohistochemical expression of matrix metalloproteinases, their tissue inhibitors, and cathepsin-D in ovarian endometriosis: correlation with severity of disease. *Fertil Steril.* 2010 Nov;94(6):2470–2472. 10.1016/j.fertnstert.2010.03.007 20385381

[109] Sotnikova NY Antsiferova YS Posiseeva LV : Mechanisms regulating invasiveness and growth of endometriosis lesions in rat experimental model and in humans. *Fertil Steril.* 2010 May 15;93(8):2701–2705. 10.1016/j.fertnstert.2009.11.024 20056200

[110] Rydlova M Holubec L Ludvikova M : Biological activity and clinical implications of the matrix metalloproteinases. *Anticancer Res.* 2008;28(2B):1389–1397. 18505085

[111] Wang J Ma X : Effects of estrogen and progestin on expression of MMP-2 and TIMP-2 in a nude mouse model of endometriosis. *Clin Exp Obstet Gynecol.* 2012;39(2):229–233. 22905471

[112] Chatterjee K Jana S DasMahapatra P : EGFR-mediated matrix metalloproteinase-7 up-regulation promotes epithelial-mesenchymal transition via ERK1-AP1 axis during ovarian endometriosis progression. *FASEB J Off Publ Fed Am Soc Exp Biol.* 2018 Aug;32(8):4560–4572. 10.1096/fj.201701382RR 29558202

[113] Liu F Zhou J Zhang X : Whole-exome sequencing and functional validation reveal a rare missense variant in MMP7 that confers ovarian endometriosis risk. *Hum Mol Genet.* 2022 Aug 17;31(15):2595–2605. 10.1093/hmg/ddac062 35288736

[114] Karamanou K Franchi M Vynios D : Epithelial-to-mesenchymal transition and invadopodia markers in breast cancer: Lumican a key regulator. *Semin Cancer Biol.* 2020 May;62:125–133. 10.1016/j.semcancer.2019.08.003 31401293

[115] Xin L Hou Q Xiong QI : Association between matrix metalloproteinase-2 and matrix metalloproteinase-9 polymorphisms and endometriosis: A systematic review and meta-analysis. *Biomed Rep.* 2015 Jul;3(4):559–565. 10.3892/br.2015.447 26171166 PMC4486806

[116] Orlichenko LS Radisky DC : Matrix metalloproteinases stimulate epithelial-mesenchymal transition during tumor development. *Clin Exp Metastasis.* 2008;25(6):593–600. 10.1007/s10585-008-9143-9 18286378

[117] Ellis K Munro D Clarke J : Endometriosis Is Undervalued: A Call to Action. *Front Glob Womens Health.* 2022 May 10;3:902371. 10.3389/fgwh.2022.902371 35620300 PMC9127440

[118] Penrod N Okeh C Velez Edwards DR : Leveraging electronic health record data for endometriosis research. *Front Digit Health.* 2023;5:1150687. 10.3389/fdgth.2023.1150687 37342866 PMC10278662

[119] Kimmelman J Federico C : Consider drug efficacy before first-in-human trials. *Nature.* 2017 Jan 30;542(7639):25–27. 10.1038/542025a 28150789

[120] Cousins FL Kirkwood PM Murray AA : Androgens regulate scarless repair of the endometrial “wound” in a mouse model of menstruation. *FASEB J Off Publ Fed Am Soc Exp Biol.* 2016 Aug;30(8):2802–2811. 10.1096/fj.201600078R 27121597

[121] Li X Zhu L Wang B : Drugs and Targets in Fibrosis. *Front Pharmacol.* 2017;8:855. 10.3389/fphar.2017.00855 29218009 PMC5703866

