# Unveiling the fibrotic puzzle of endometriosis: An overlooked concern calling for prompt action

**DOI:** 10.12688/f1000research.152368.3

**Published:** 2024-12-03

**Authors:** Megha M Anchan, Guruprasad Kalthur, Ratul Datta, Kabita Majumdar, Karthikeyan P, Rahul Dutta

**Affiliations:** 1 Division of Reproductive Biology, Department of Reproductive Science, Kasturba Medical College, Manipal, Manipal Academy of Higher Education, Manipal, Karnataka, 576104, India; 2 Nova IVF fertility, Guwahati, Assam, India; 3 Gauhati Medical College & Hospital IVF centre, Bhangagarh, Gauhati Medical College, Assam, 781032, India; 4 Department of General Surgery, Government Kallakurichi Medical College, Government Kallakurichi Medical College, Kallakurichi, Tamil Nadu, India

**Keywords:** Endometriosis, pelvic pain, etiology, animal model, Epithelial-mesenchymal transition, fibrosis

## Abstract

Endometriosis is a benign, estrogen-dependent, persistent chronic inflammatory heterogeneous condition that features fibrotic adhesions caused by periodic bleeding. The characteristic ectopic lesions are marked by a widely spread dense fibrotic interstitium comprising of fibroblasts, myofibroblasts, collagen fibers, extracellular proteins, inflammatory cells, and active angiogenesis. Fibrosis is now recognized as a critical component of endometriosis because of which current treatments, such as hormonal therapy and surgical excision of lesions are largely ineffective with severe side effects, high recurrence rates, and significant morbidity. The symptoms include dysmenorrhea (cyclic or noncyclic), dyspareunia, abdominal discomfort, and infertility. The significant lack of knowledge regarding the underlying root causes, etiology, and complex pathogenesis of this debilitating condition, hinders early diagnosis and implement effective therapeutic approaches with minimal side effects presenting substantial hurdles in endometriosis management. Emerging research offer a close relationship between endometriosis and fibrosis, which is believed to be tightly linked to pain, a primary contributor to the deterioration of the patient’s quality of life. However, the underlying pathophysiological cellular and molecular signaling pathways behind endometriosis-associated fibrosis are poorly addressed. The available experimental disease models have tremendous challenges in reproducing the human characteristics of the disease limiting the treatment effectiveness. Future translational research on the topic has been hindered by the lack of an adequate fibrotic model of endometriosis emphasizing the necessity of etiological exploration. This review article focuses on recent developments in the field and highlight the necessity for novel fibrotic models for early diagnosis, a better understanding the disease’s etiology and develop effective anti-fibrotic treatments. By addressing these knowledge gaps, we want to open fresh avenues for a thorough investigation and extended research in the field of endometriosis.

## Introduction

Endometriosis is an estrogen-dependent chronic inflammatory disorder resulting from the implantation of viable endometrial, epithelial, and stromal cells (lesions) outside the uterus and is often associated with infertility. ^1^ The condition affects at least 10% (~247 million) of women worldwide, with Asian women reporting the highest prevalence, with over ~42 million girls and women from India, ^2, 3^ which can negatively affect the outcome of IVF treatments. ^4, 5^ Endometriosis can result in severe dysmenorrhea, dyspareunia, and menorrhagia; exacerbates pelvic/abdominal pain; and eventually leads to infertility due to considerable damage to the structure and function of reproductive organs, even compromising the entire body system through the accumulation of fibrotic tissue. ^6^ The diagnosis can take 4 to 11 years due to difficulties in classifying and identifying the disease and its peculiar symptoms, as well as a lack of diagnostic indicators. ^7^ According to Maddern et al., endometriosis has a significant effect on a person’s quality of life, reproductive health, and society at large. ^8^ Currently, the most widely recognized theory explaining how endometriosis begins is “Sampson’s theory”, which holds that the misplaced viable endometrium-like tissue is transferred onto the pelvic peritoneum by retrograde menstruation via the fallopian tubes. ^9^ Even after several decades of research, the etiology is still unclear and depends on a few key theories and assumptions, such as retrograde menstruation theory, embryonic remnants, coelomic metaplasia, immune dysfunction, inflammation, oxidative stress, hormones, dysfunctional apoptosis, the microbiome, metabolomics, endocrinology, and genetic expression differences, which fail to explain its pathophysiology ^2, 9^ adequately. Although retrograde menstruation occurs in 90% of reproductive-age women, only 10% develop endometriosis, indicating that additional relevant factors contribute to disease onset and progression within the peritoneal cavity. This disparity suggests that complex networks contribute to the emergence of this challenging condition. ^10, 11^ This entails understanding how cells from the normal lining of the uterus find atypical locations, multiply excessively, escape immune and apoptotic processes, and acquire the necessary blood supply and nutrients that ultimately result in the formation of aberrant fibrotic lesions that contribute to the distinctive symptoms triggered by endometriosis, including excruciating pain and infertility. ^12^ None of the available theories fully capture the intricacies of fibrotic endometriosis, emphasizing the need for additional studies to identify the pathophysiology of endometriosis. ^13^ The production of fibrotic tissue comprising fibroblasts, myofibroblasts, collagen fibers, and inflammatory cells is increasingly recognized as a crucial element contributing to disease severity, resistance to treatment, and high recurrence rates. This paucity of understanding of the molecular and cellular mechanisms encouraging fibrotic endometriosis provides an important barrier to the development of effective diagnostic tools and therapeutic strategies. ^14^ Moreover, the American Society of Reproductive Medicine (rASRM) categorization score approach does not account for pathology-based staging on the basis of fibrosis, which includes epithelial-to-mesenchymal transition (EMT), mesenchymal-to-epithelial transition (MET), or smooth muscle metaplasia (SMM). This means that patients with fibrotic characteristics and adhesions may fail to obtain a reliable diagnosis. ^15^ Integrating fibrosis-specific indicators into diagnostic standards should increase the reliability of endometriosis diagnosis and staging, allowing for more targeted and successful treatment options. ^16^ The formation, invasion, and angiogenesis of fibrotic ectopic lesions are also associated with disrupted immunoregulatory processes and a variety of inflammatory markers, including immune cells, cytokines, chemokines, matrix metalloproteinases, and other components associated with the immune system. ^17, 18^ Thus, a thorough understanding of the mechanisms underlying the origin and evolution of fibrotic endometriosis is crucial for managing and evaluating the risks associated with this condition. This review highlights the critical need to investigate and outline the molecular drivers of fibrotic endometriosis. In this review, we intend to address these gaps by providing a detailed understanding of the role of fibrosis in endometriosis, evaluating existing endometriotic models, identifying significant research gaps, and proposing new directions for exploration. We emphasize that an improved understanding of fibrotic pathways in endometriosis may aid in the development of novel therapeutics that target fibrosis, thus improving the prognosis of patients.

## Method

We conducted an electronic database literature search of PubMed and Google Scholar for published research articles on endometriosis and endometriotic animal models. The search terms “endometriosis”, “endometriosis mouse model”, “primate model of endometriosis”, “endometriotic patients”, and “endometriosis-associated fibrosis” were used. Articles with thorough experimental data and definitive results were considered for inclusion; those with inconclusive research findings were eliminated. We incorporated clinical trials, surveys of endometriosis-affected women, and observational and experimental studies, including animal studies, as references. Research written in languages other than English was not considered. All the graphics were prepared via Biorender software ( BioRender.com).

## Literature review

### Endometriotic models: Importance of addressing gaps in preclinical animal models

**Figure 1.**
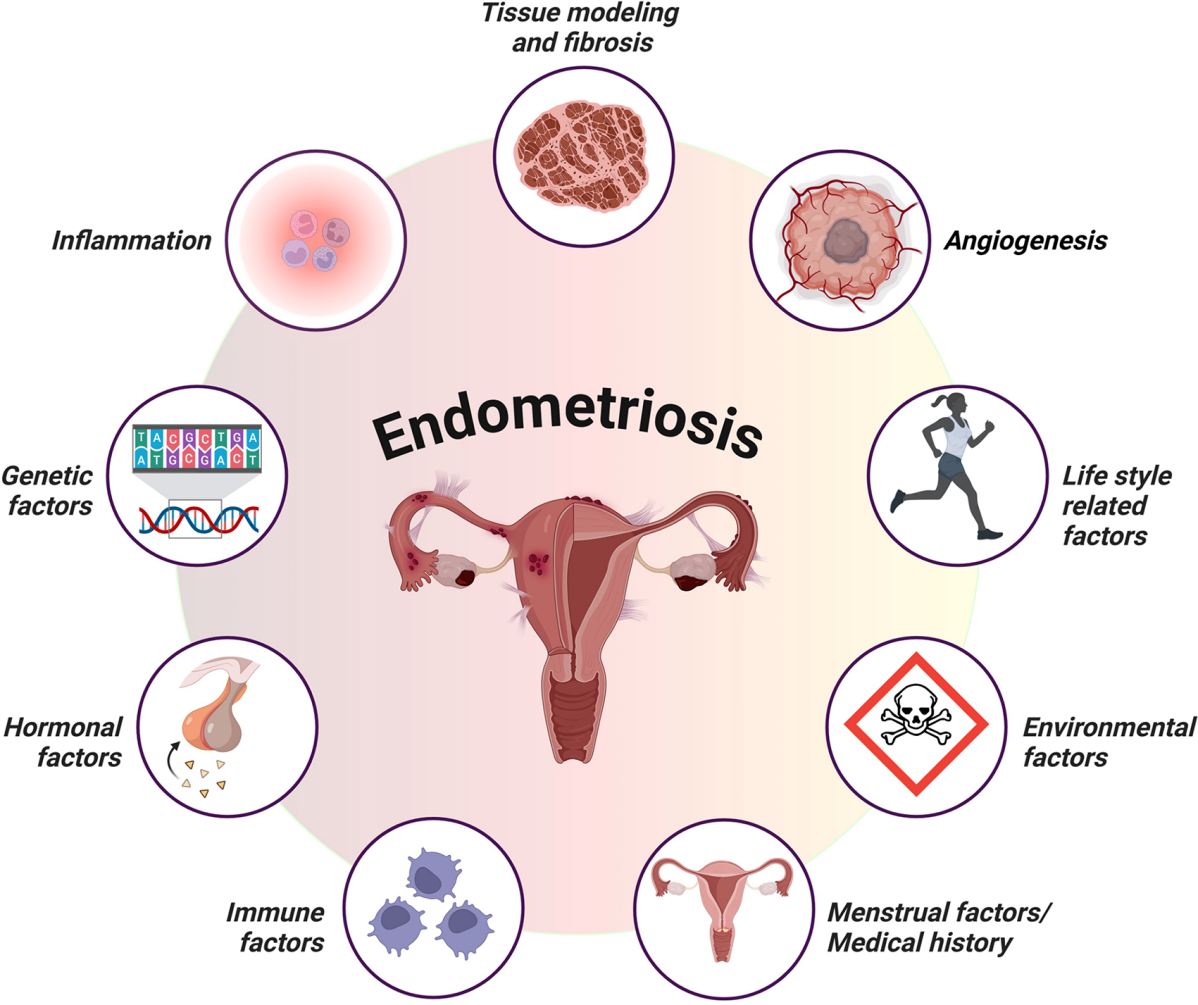
Schematic representation of key factors contributing to the development and progression of endometriosis. The illustration highlights the interplay between genetic factors, hormonal imbalances, immune dysfunction, and inflammation, including lifestyle-related and environmental factors. These factors collectively influence lesion establishment, persistence, and growth, providing a comprehensive overview of the multifactorial nature of endometriosis pathophysiology (created with Biorender.com).

Owing to the unavailability of definitive treatments and the limited understanding of endometriosis, researchers have attempted to develop animal models to provide insights into its causes and to identify novel therapeutic targets. The most extensively studied animal models for endometriosis include autologous or syngeneic rodent models, xenotransplantation of human endometrial tissue into immunodeficient mice, and, to a lesser extent, owing to ethical considerations and expensive costs, nonhuman primate models. ^19^ The most significant distinction between these models is that endometriosis develops spontaneously in nonhuman primates but not in rodents. ^19^ According to Greaves et al., endometriosis is currently being studied via two basic approaches: human-based *in vitro* samples and experimental *in vivo* animal models. ^20^ The first type involves experimental *in vitro* research using tissue biopsies and fluids obtained from resected lesions or aspiration biopsies, such as endometrial and peritoneal explants, endometriotic cell lineages, primary endometrial stromal cells, endometrial stem cells, and immune cells. ^21^ *In vivo* animal models are essential for assessing drug candidates and preclinical trial testing. Our knowledge of the early phases of disease development, including the effects of the peritoneal microenvironment, inflammatory responses, and steroid responsiveness, has improved because of these models. ^22^ However, for a variety of reasons, it has been difficult to create *in vitro* or *in vivo* models that accurately replicate the features found in endometriotic patients. Endometriosis is complex, multifactorial, and heterogeneous, and the uncertainty underlying its onset further complicates the development of reliable models. Second, the disease manifests in several forms, including peritoneal, deep infiltrative lesions, and ovarian endometriomas, each exhibiting distinct pathological characteristics. ^23^ Finally, endometriosis cannot be effectively characterized based on a single pathophysiological mechanism. Additionally, this condition is connected with genetic, ^24^ immunological, ^25^ environmental, ^26, 27^ and hormonal changes, such as progesterone resistance ^28^ and estrogen reliance, ^29^ further challenging the establishment of acceptable animal models ( Figure 1). Additionally, most animal models fail to adequately mimic crucial characteristics of human endometriosis, such as persistent chronic fibrosis. These limitations hinder the successful translation of research findings to human disease settings. These findings emphasize the need for a higher-fidelity mouse model that better portrays the complex pathophysiology of endometriosis in humans. ^19, 30^ Despite these constraints, progress has been made in the development of representative endometriosis models, but these existing models have major limitations, emphasizing the need for additional research to bridge this gap in knowledge.

Considering all of these factors and all the possible limitations of rodent models, researchers have focused on nonhuman primates (NHPs), such as baboons ( *Papio anubis*) and rhesus monkeys, because they spontaneously develop endometriosis and menstruate in a cyclic pattern. Interestingly, even in NHPs, surgically induced endometriosis reduces fertility, much like it does in humans. Cynomolgus monkeys ( *Macaca fascicularis*) with moderate or severe endometriosis have been shown to have lower rates of fertilization and pregnancy following surgery. ^31^ In addition, subfertility due to endometriosis is tied to disease stage in baboons. ^32^ The work by Nishimoto-Kakiuchi et al. ^33^ presents novel and crucial insights from a nonhuman monkey for translational research in endometriosis, where they carefully examined screening, diagnosis, staging, and monitoring in a population of cynomolgus monkeys. They proposed a robust methodology that has the benefit of employing an animal model with a lower body size than baboons do, making it easier to monitor and handle in an experimental setting. However, the major limitation of this model is the reduced incidence rate of endometriosis, which is only 28.7%. ^33^ In this context, NHP models appear to be the best model animals for endometriosis research owing to their phylogenetic, anatomical, and reproductive similarities to humans. Moreover, they experience spontaneous endometriosis, as observed in humans. ^34, 35^ However, in some species ( *Papio anubis*), the menstrual period is nearly every 4 weeks, corresponding to that of humans. Indeed, the diagnosis of spontaneous disease in NHP models is problematic, as a substantial animal number is necessary for induction, and there is a lack of accurate noninvasive tools for early detection. ^36^ Nonetheless, NHP models are useful for studying the etiology, development, and progression of the disease and possibly evaluating the efficacy of drugs. However, more research is needed to confirm the effectiveness of the “biological response,” which is correlated with endometriosis and its symptoms. This could lead to improved diagnostic accuracy and early detection in NHP models, which would be in line with the main goals of clinical endometriosis research in humans.

### Fibrotic endometriosis overview: knowledge gaps and challenges

Endometriosis is characterized by the persistent occurrence of fibrosis and myofibroblasts within endometriotic lesions, which play critical roles in disease development, making fibrosis a molecular hallmark of endometriosis. ^37^ Notably, significant scarring is commonly linked to endometriosis. ^37^ Although the initial onset of endometriosis is associated with the existence of endometrial stroma and glands in abnormal locations, the endometrial components are often soon replaced by fibrotic and smooth muscle components. ^38^ For example, rectovaginal nodules display glandular epithelium embedded deeply within fibromuscular tissue devoid of any surrounding stroma. ^39^ Similarly, in 40% of ovarian endometriomas, the endometrial epithelium is not detected, and the interior of the cyst is covered solely by fibrotic tissue. ^40^ Despite being a crucial pathological feature of this disease, pelvic adhesions generally lack any endometrial components. ^41^ These adhesions contribute to the pathology of some common symptoms of endometriosis, including chronic pelvic pain, deep dyspareunia, and infertility presumably aggravated by these fibrotic formations. ^41^ The process by which endometriosis progresses to a malignant condition remains unknown. However, continuous inflammation, immunological dysregulation, and fibrosis, most likely caused by iron-induced oxidative stress, may lead to genetic changes, which may lead to malignant features. ^14, 42^ Fibrosis is believed to be linked to pain, which is the disease’s most common symptom and the principal cause of a patient’s poor quality of life. ^43^ Thus, understanding the underlying mechanisms will help to understand why the morphological characteristics of the disease do not match the degree and nature of fibrosis-related pain reported. ^44^

Fibrotic tissue is characterized by excessive development of extracellular matrix (ECM) components inside and around inflamed or damaged tissue, and it is a typical and significant phase of tissue repair in all organs. Fibrosis involves activated platelets, macrophages, and myofibroblasts, which results in increased collagen deposition. ^45^ Fibrosis and smooth muscle metaplasia are two of the main characteristics of endometriosis in women with fibrosis surrounding endometriotic tissue, and the degree of fibrosis is correlated with the severity of smooth muscle metaplasia. ^46^ Endometriotic lesions are thought to be “wounds” that undergo repeated tissue injury and repair (ReTIAR), leading to TGF-β1/Smad3-mediated EMT and ultimately resulting in fibrosis as the lesions progress. In essence, regardless of location or subtype, all endometriotic lesions are recognized to be identical to wounds that undergo ReTIAR, ultimately resulting in the fibrotic appearance of both ovarian endometriomas (OMAs) and deep infiltrating endometriosis (DIE). ^47, 48^ This process enables solitary cells to pass through the basement membrane, grow invasively, and metastasize by both intra- and extravasation. ^9^ However, if the underlying mechanisms are known, they may explain why the disease’s morphological characteristics do not match the extent and nature of fibrosis-induced pain sensations. ^49^ However, there is a paucity of information on the development of preclinical models that define clinically effective endpoints such as chronic fibrosis. Additionally, Modi et al.’s mouse model of endometriosis revealed considerable inflammation but lacked histological signs of fibrosis, with neither EMT nor fibrosis commonly reported in such models. ^50^ Consequently, studies on the molecular pathways associated with fibrosis or possible targets for therapeutic intervention for fibrosis in endometriosis have been stopped because of the unavailability of an animal model of endometriosis. ^50, 51^ Furthermore, 50–70% of drugs that have advanced to phase II and III clinical trials are unable to show efficacy, indicating the insufficiency of current disease models in the exploration of critical biological processes. ^52^ These findings suggest that there are no reliable animal models for examining significant cellular processes associated with endometriosis. Given the chronic nature of the disease, we believe that chronic fibrosis may play a major role in the progression of endometriosis, potentially leading to fibrotic adenomyosis. In summary, an optimal model for understanding endometriosis that mimics the cellular and pathophysiological processes and clinical behaviors observed in human patients, notably fibrosis coupled with invasion and metastasis, is needed. Despite these limitations, considerable improvements have been made in the development of endometriotic models for fibrosis-based research studies.

### Primate model of endometriosis

Endometriosis is challenging to eliminate because of the inadequate understanding of its genesis and pathophysiology. Controlled experimental investigations on humans are limited because assessing disease prevalence and development necessitates numerous laparoscopies, which are challenging for multiple reasons. Although endometriosis occurs spontaneously in humans, human investigations have been limited for ethical and practical reasons, with one of the primary reasons being the difficulty of studying the disease. As a result, understanding the etiological mechanisms of this disease requires the use of an appropriate animal model. The evidence for fibrosis has primarily been derived from *in vitro* experiments on human endometriotic tissues and *in vivo* studies on nonhuman primates, which are potential candidates for research because of their anatomical and physiological resemblance to humans. ^34^ Endometriosis is recognized to occur exclusively in menstrual animals, including nonhuman primates, such as rhesus macaques ^53^ and baboons, ^54^ because their endometrial morphology, physiology, and menstrual cycle are nearly identical to those of women. ^54^ Baboons are capable of developing spontaneous endometriosis, which makes them particularly relevant models for investigating this disease. ^55^ Two types of endometriotic models have been established in baboons. Spontaneously ^34^ and experimentally generating endometriosis via autologous endometrial transplantation. ^56, 57^ Moreover, induced endometriosis in NHPs closely resembles spontaneous endometriosis that develops in women. ^58^ It was also reported that iatrogenically induced retrograde menstruation might lead to the onset of endometriosis, validating the concept of Sampson. Endometriosis was experimentally generated in rhesus macaques via surgical diversion of the cervix into the abdomen. However, endometriosis has been identified in only 50% of animals. ^59^ The first baboon experimental model of nodular endometriosis was established by Donnez et al. in 2023 for the exploration of deeper nodular lesions as well as invasion events connected with nodular lesions. ^60^ Frequent surgical interventions, however, have been shown to provoke the spontaneous growth of endometriotic lesions and could modify the functionality of the endometrium. ^61^

According to Zhang et al., a baboon endometriosis model demonstrated the progressive nature of EMT, FMT, and fibrosis. This led to the expansion of fibrosis from minor fibrosis at three months to highly fibrotic lesions at twelve months after endometriosis induction. This strongly suggests the progressive nature of the disease. ^47^ Additionally, histological analyses revealed that fibrosis in baboon endometriosis closely mirrors that observed in human cases, making it an appropriate model for investigating disease progression and treatment outcomes in patients with fibrosis. Donnez et al. discovered altered morphology, elevated mitotic activity, and fewer adhesion molecules in invasive glands associated with induced nodular endometriosis, implying that cell migration is involved in the process of invasion of deep fibrotic endometriotic lesions generated in a baboon model. ^62^ A model of iatrogenic deep nodular endometriotic lesions was developed to construct an experimental model of replicating human deep nodular fibrotic lesions. ^60^ Deep nodular endometriotic lesions created in the baboon closely mirror spontaneous deep-infiltrating nodules in invasive and noninvasive lesions. ^60, 63^ A recent investigation in baboon models indicated that the overexpression of IL-6 enhances the expression of fibrotic factors, inducing fibrosis via the TGF-β signaling pathway. These findings in baboons closely match those in humans with endometriosis reinforcing the concept that fibrosis is a critical component of the disease course. ^64^

### Limitations of nonhuman primate models in endometriosis research

The use of NHP in endometriosis research is not free of potential drawbacks or limitations. First, the low incidence rates, i.e., 4.8% and 20.7%, of spontaneous and induced endometriosis, respectively, demonstrate that baboons can cleanse and regenerate their peritoneum, which may decrease the significance of the model. ^65^ In contrast, in rhesus monkeys, ^66, 67^ the significance of peritoneal cysts in endometriosis pain and discomfort has not been investigated. The cynomolgus monkey ^33, 68^ has been described, with the limitations that deep lesions are difficult to diagnose and that time course changes in the condition are not investigated. Other challenges include a relatively small cohort of endometriotic animals for experimentation, an extended period of gestation for fertility research, a longer duration to develop endometriotic lesions, the difficulty of dealing with conscious baboons, and the high cost of experimentation and maintenance, which require larger doses of medications, specialized infrastructure, logistics, and special training for handling these animals. It is also perceived to be ethically sensitive and expensive. ^57, 69^ Consequently, rodent models are commonly used for preclinical efficacy testing for therapeutic interventions owing to their reduced costs and ease of handling.

### Rodent models of endometriosis

Preclinical modeling is crucial for investigations of disease pathogenesis, biomarker development, and preventative and therapeutic discovery. This is particularly true for complex conditions, such as endometriosis, where nonsurgical diagnostic techniques to allow longitudinal clinical study designs remain unavailable. Rodents are frequently employed as preclinical models in biomedical research since they are molecularly well-annotated species. This permits researchers to utilize different interrogative strategies to dissect multifactorial disorders. Their usefulness for examining the molecular foundations of disease pathogenesis lies in the simplicity of genetic modifications and their ability to target potential genes for specialized study. ^70^ Additionally, given the lack of accessibility and high costs related to nonhuman primates, rodents offer a convenient and inexpensive alternative for researching the origins and course of disorders such as endometriosis. However, because research facilities for primates/nonhumans are limited, nonprimate experimental animal species, such as mice or rats, are regarded as suitable first-line tools for researching the origin of this puzzling disease. Endometriosis is characterized by the recurrent development of new lesions with each menstrual cycle and the advancement of preexisting lesions. Therefore, additional research is needed to understand the natural course and gradual development of endometriosis lesions. ^71^ There is evidence of gradual lesion clearing, but only a small number of studies using mouse models of endometriosis have investigated disease induction and regression. ^71, 72^ While rodent models have been valuable for researching the disease, especially its pathophysiological and molecular underpinnings, gaps exist in understanding fibrotic lesion progression. Most importantly, owing to the ethical limits of frequent laparoscopic screening of endometriotic patients, rodent models provide essential longitudinal investigations to increase the translational value of preclinical findings. ^71^

Mice are the most popular experimental animal models because of their ease of gene manipulation, availability, easy handling, tissue similarity *in vivo*, small size and large litter, which make them cost-effective, and their relatively short gestation, which allows transgenerational examination. ^22^ On the basis of the available research publications, two types of mouse models have been successfully used to implant endometriotic lesions. The first approach involves suturing, where human endometriotic implants are surgically autotransplanted into the peritoneum of immunocompromised mice. ^73– 75^ The second approach involves the intraperitoneal or subcutaneous implantation of autologous uterine segments into the peritoneum of recipient mice from a syngeneic donor. ^76– 78^ Mouse models have aided in investigating several aspects of this disorder, such as early disease phases, ^79^ steroid hormone involvement, ^80^ host inflammatory mechanisms, ^81, 82^ oxidative stress, ^83, 84^ neuroangiogenesis, ^76^ and infertility. ^85^ While these methods have enhanced our understanding of disease pathways, challenges persist. For example, immunocompetence is a difficulty when employing human uterine tissue or human endometriotic tissue in a mouse model. Immunocompromised mice may not reflect the environment within the human peritoneal cavity, and the outcomes of the experiment may not correctly reflect disease onset. ^86^ In ovariectomized mouse models generated with exogenous estrogen, estrogen reliance drives lesion progression in endometriosis; however, these models add surgical factors and off-target effects. Because endometriosis mirrors natural hormonal cycles, hormonally intact mice offer a more realistic representation. ^75^ However, mice, like other members of the rodent family, typically do not menstruate and hence do not develop endometriosis spontaneously. They also have a closed reproductive system and are highly fragile with respect to dietary needs. Consequently, earlier studies modeling endometriosis utilizing mice required stimulation of menstruation or endometrium transplantation for the development of endometriotic lesions. ^70^ Hence, there are publications that claim that these lesions do not adequately mirror real endometriosis, as they lack features such as persistent fibrosis. ^87^

On the other hand, rats can produce only superficial lesions, which are the most fundamental and possibly least clinically significant types of lesions. Many studies using rodents as a model for endometriosis have investigated the gene expression patterns of ectopic tissue deposits in rats in an attempt to correlate them with human endometriotic lesions. Chronic inflammation, angiogenesis, and extracellular matrix remodeling are common pathways. ^86– 88^ While some aspects of the disease are replicated in the rodent model, all the modifications involve suturing uterine fragments (endometrium plus myometrium) to different sites, which does not accurately represent the formation of lesions from those shed endometrial tissue or the dissemination of menstrual tissue into the peritoneum. Notably, particularly in terms of understanding its pathophysiology and treatment options, the current rodent models have not been successful in yielding findings that apply to human endometriosis. The inability of any study to recreate fibrotic endometriotic lesions may account for the failure of rat models to yield data relevant to the pathophysiology and treatment of human endometriosis. This situation demonstrates that the preclinical animal studies that have been established are not transferable. ^89^ Therefore, fibrosis, a mostly disregarded component of human endometriosis, should be taken into consideration. ^89, 90^ We reviewed the existing mouse models in the context of the optimal parameters found in well-evidenced pathophysiologic aspects identified in endometriosis ( Table 1). Collectively, these models have yielded critical insights and advanced the replication of the molecular characteristics of this disease. Owing to their ability to model chronic fibrosis, mouse models constitute a powerful resource for translational research in endometriosis. Therefore, developing novel rodent models that mirror the continuous fibrotic process observed in endometriotic patients is essential for improving our understanding of this disease. Emerging research has recently focused on the role that fibrosis plays in clinical-grade endometriosis. On the other hand, little is known about fibrosis treatment strategies. Therefore, developing a fibrotic mouse model of endometriosis, elucidating the regulatory processes underlying fibrosis in endometriosis, and identifying more precise specific biomarkers for this disease are critical. These markers can also be utilized to find effective therapeutic targets and identify endometriosis in its early phases. The successful translation of potential discoveries obtained in a preclinical model to humans is dependent primarily on model fidelity. To mimic the fibrotic scarring observed in endometriosis, many endometriotic fibrotic mouse models have been developed ( Table 1).

**Table 1.** Summary of available mouse models of endometriosis, demonstrating the presence of fibrotic markers. The table includes details on the type of model, approach used for model development, and specific fibrotic markers and pathways explored. This analysis emphasizes the heterogeneity in fibrotic marker expression across different models and provides insights into their relevance for researching the fibrotic elements of endometriosis.

Experimental model	Induction method	Fibrotic genes involved	Mechanism	Inflammatory response	References
BALB/c	Surgical method	TGF-β, COL1A1 and COL3A1, α-SMA	Platelet activation contributing to EMT, FMT, and SMM	Activation of TGF-β1	^93^
Swiss nude mice	Transplantation of human endometrial tissues	α-SMA, COL1A1, fibronectin, CTGF	Cell proliferation and migration Enhanced collagen gel contraction Wnt/β-catenin signaling	Wnt/β-catenin interaction with TGF-β1	^94^
C57BL/6	Transplanting shed endometrial tissue from female donor mice into recipient mice	Fibronectin, COL1A1	Shed endometrial tissue as a key source of pro-inflammatory mediators thereby driving fibrosis	IL-6, TNFα, CCL2 and CCL5	^76^
BALB/c	Intraperitoneal injection of uterine fragments from donor mice	α-SMA, FSP-1/S100A4, Desmin, vimentin	EMT, FMT, SMM, MMT, EndoMT	SP and CGRP sensory nerve-derived inflammatory mediators	^95^
BABL/c nude mice	Transplanting endometrial tissue into the peritoneal cavity of mice	Fibronectin, ColA1, α-SMA, and CTGF	Paracrine signaling of eMSCs	Thrombospondin 4	^96^
Swiss nude mice	Implanting pieces of autologous endometrial tissue into the peritoneal cavity of the mice	A-SMA, Col-I, FN and CTGF	TGF-β signaling	TNF-α, IL-6	^97^
BALB/c	Intraperitoneal injection of human eutopic endometrial tissue	Collagen I, α-SMA, and CTGF	CTGF signaling	-	^98^
C57BL/6	Heterotransplantation with immortalized human endometrial cells	α-SMA, COL1A-I, FN and CTGF	mTOR signaling	-	^99^
C57BL/6	Donor endometrial tissue fragments transplanted into the recipient	α-SMA, COL1AI, TGF-β1	Platelet activation and fibrosis	CD41	^100^
Athymic nude mice	Subcutaneous injection of proliferative endometrial fragments	α-SMA, COL1A1, CTGF, FN	Wnt/β-catenin pathway	TGF-β1	^101^

Alpha-smooth muscle actin (α-SMA), connective tissue growth factor (CTGF), fibronectin (FN), transforming growth factor beta (TGF-β), COL1A1 and A3 (collagen types 1 and 3), epithelial-to-mesenchymal transition (EMT), fibroblast-to-myofibroblast transdifferentiation (FMT), smooth muscle metaplasia (SMM), tumor necrosis factor α (TNFα), monocyte chemoattractant protein chemokine ligands 2 and 5, fibroblast-specific protein 1 (FSP1), S100 calcium-binding protein A4 (S100A4), TNF-α (tumor necrosis factor-alpha) and IL-6 (interleukin-6), the mesothelial-mesenchymal transition (MMT), the endothelial-mesenchymal transition (EndoMT), endosome-derived mesenchymal stem cells (eMSCs), and mammalian target of rapamycin (mTOR).

### Limitations of rodent models in endometriosis research

Endometriosis is termed the ‘missing disease’ because of its ambiguous etiology and discrepancies in its origin, diagnosis, and treatment. ^102^ Despite a recent surge in endometriosis research, the underlying pathobiology of the disease remains poorly known, implying that animal models of the disorder are crucial for future studies in this field. This ambiguity highlights the need for animal models that precisely mimic human endometriosis and elucidate its conditions, which can provide a basis for subsequent research. ^103^ One of the most significant obstacles in endometriosis research is the lack of reliable mouse models that characterize the manifestations of this condition in humans. ^104^ Ideally, a disease model should mirror human disease, allowing researchers to investigate the effects of intrinsic (e.g., genes) and extrinsic (e.g., environment) factors on disease progression. Many previous studies linked fibrosis secondary to the development of endometriosis, and there has not been much research on fibrosis as a primary focus. ^15, 105^ Research from animal models revealed that a percentage of women receiving hormone therapy in human trials do not respond to these drugs ^105^ and require surgical lesion removal to alleviate symptoms. Women may have endometriotic lesions that have progressed to a fibrotic state by the time they seek medical attention, rendering treatment ineffective. This highlights the urgent need to develop an *in vivo* model that can effectively mimic the development and characteristics of human endometriosis, opening avenues for more effective treatments and a deeper understanding of this disease. These findings will also facilitate the understanding of the connection between the origin of fibrosis in endometriosis, existing medical care, and potential targets for therapy. In conclusion, although the literature emphasizes the importance of fibrosis in the course of endometriosis, gaps remain in understanding the underlying genes and pathways related to the fibrotic aspect of the disease. While existing rodent models highlight certain factors, such as inflammation and immune dysregulation, they often overlook fibrosis, thus poorly reflecting the complexity of the disease. In addition, these models insufficiently depict the degree of severity, traits, and drivers of fibrosis in clinical human endometriosis. Additionally, the complex interplay of signaling mechanisms that promote lesion formation in a fibrotic milieu remains inadequately studied. These limitations highlight the demand for improved fibrotic-based animal models that accurately replicate the disease and offer an in-depth investigation of fibrotic pathways. Although studies have provided insight into genes that contribute to fibrosis in endometriosis, further exploration of the complicated signaling networks underlying this disease remains important. This gap highlights the necessity for future investigations employing advanced methodologies such as knockout animal models, high-throughput RNA sequencing, and omics techniques. These techniques provide greater insights into the mechanisms of fibrotic markers and assist in confirming their function in endometriosis growth, providing strong evidence for the creation of medications that delay, terminate, and reverse fibrosis advancement and benefit endometriotic patients. Additionally, many of the current animal models of endometriosis can be further enhanced by altering them to allow noninvasive *in vivo* monitoring of lesion size, as this approach is desirable for preclinical models of endometriosis.

### Human experiment details

After years of relentless advocacy from individuals affected by the condition, endometriosis is gradually gaining increased attention, as evidenced by an increase in research, particularly large-scale controlled human trials and meta-analyses, which have the potential to significantly increase awareness of the condition and its management. Except for several NHPs, animals do not develop endometriosis spontaneously; hence, *in vitro* models employing human tissues have been employed to research the pathophysiology of this medical condition ( Table 2). The majority of currently known *in vitro* models utilize several cell or tissue types, including endometriotic cell lines as monolayer culture models, human primary endometrial epithelial and stromal cells, endometrial stem cells, endometrial explant cultures, and coculture models with peritoneal cells and immune cells. ^106– 108^ Each model exhibits unique characteristics and functions and is able to illustrate one or more components of the process of endometriosis. These models are helpful and can be used to explore the origin of endometriosis and the underlying mechanisms of this condition in depth and assist investigators in selecting relevant models for their research. ^21^ In recent years, researchers have developed different *in vitro* models of varying complexity that provide helpful tools to unravel the processes involved in the etiology of endometriosis. Most cell culture methods are maintained in 2D settings; however, more advanced 3D models are becoming more prevalent to improve the specific endometriosis milieu. They offer the chance to examine endometriotic cell connections with surrounding cells and analyze unique cross-talk between cells. ^106^ Patient-obtained tissues of ectopic and eutopic endometria or biopsy samples from endometriotic cysts and fluids from women with and without endometriosis undergoing laparoscopy for diverse research goals are being used. However, the protocol variation employed for collecting, processing, and storing samples certainly restricts the compilation and repeatability of data produced at different research institutions.

**Table 2.** Overview of *in vitro* studies on endometriosis tissues demonstrating the presence of fibrotic markers. The table outlines the type of endometriosis tissue used, specific fibrotic markers evaluated, and key pathways. This compilation highlights the contributions of *in vitro* systems in unraveling the molecular mechanisms underlying fibrosis in endometriosis.

Sample type	Fibrosis associated markers	Pathway	References
OE/Ovarian cysts	Collagen I, α-SMA, Fibronectin	TGF-β1/Smad signaling	^109^
DIE or OE	α-SMA, collagen I, CTGF	Wnt/β-catenin signaling	^110^
DIE with or without OE	AKT and ERK	AKT and ERK signaling	^111^
Endometriotic ectopic implants	α-SMA, collagen I	ADAM17/Notch signaling	^112^
OE or DIE	α-SMA, N-cadherin, Vimentin, Snail, Slug, Desmin, Fibronectin, LOX, PAI1	TGF-β1, PDGF, Wnt/β-catenin	^113^
OE	α-SMA, COL1A1, CTGF, FN	mTOR signaling	^99^
OE	GLI3, HOXA10 and HOXA9, MAPK8 (JNK1), GATA2, ETS2	TGF-β signaling, MAPK signaling pathway, FoxO signaling pathway	^114^
Endometriomas	FAK, MCP1, TGF-β1, α-SMA	PI3K/Akt and focal adhesion kinase (FAK) pathways	^115^

OE - Ovarian endometrioma, DIE - Deep infiltrating endometriosis, Transforming Growth Factor β1 (TGF-β1) Pathway, Platelet-Derived Growth Factor (PDGF) Pathway, Wnt/β-catenin Pathway, α-SMA (alpha-Smooth Muscle Actin), COL1A1 (Collagen Type I Alpha 1 Chain), CTGF (Connective Tissue Growth Factor), FN (Fibronectin), rapamycin (mTOR) signaling, GLI3: GLI Family Zinc Finger 3, HOXC8, HOXA9 and A10: Homeobox C8 and A10, MAPK8: Mitogen-Activated Protein Kinase 8 (also known as JNK1), ETS2: ETS Proto-Oncogene 2, Transcription Factor, GATA2: GATA Binding Protein 2, FAK (Focal Adhesion Kinase), TFAP2C: Transcription Factor AP-2 Gamma, PRDM1: PR/SET Domain 1 (also known as BLIMP-1).

According to Fan 2020, in addition to studying the origin and mechanisms behind fibrosis in endometriosis, *in vitro* models are a viable tool for investigating therapeutic innovations for the management of endometriosis. ^21^ The idea that endometriosis is a fibrotic disease has prompted studies to explore how myofibroblasts differentiate and how fibrosis develops in endometriotic lesions. This will lead to the development of new models that can be used to study endometriotic fibrosis. Thus, future studies should concentrate on myofibroblast differentiation and activity in endometriotic lesions. Advances in *in vitro modeling* technology could revolutionize the study of endometriosis pathophysiology and allow the discovery of new targets to develop effective treatment approaches.

### Interplay of EMT and MMPs in endometriosis

Endometriosis is a common benign gynecological disease with a high propensity for migration and invasion. The cell-to-cell or cell-ECM connections allow the cells to migrate, invade, and proliferate in new locations. MMPs are linked to adhesion, invasion, and the severity of endometriosis. These findings indicate that MMPs play a role in extracellular matrix remodeling, which is necessary for the development of ectopic endometriosis lesions. ^116^ They are also significantly more abundant in the endometrial and peritoneal fluid of endometriosis patients. ^117, 118^ Matrix metalloproteinases (MMPs) are a family of enzymes that are mostly found in the functional layer of the endometrium. They are secreted by resident immune cells and stromal fibroblasts, which facilitate the remodeling of the extracellular matrix, including collagen, elastins, and other glycoproteins, and endometrial disintegration during menstruation. Tissue inhibitors of matrix metalloproteinases (TIMPs) are endogenous antagonists that reduce MMP overexpression, and ovarian steroid hormones are known to control MMP activity. ^119^ EMT is a process in which epithelial cells lose the polarized structure of the cytoskeleton and acquire the enhanced motility of mesenchymal cells. These modifications are considered necessary for the original formation of endometriotic lesions. While fibrosis has been recognized as a prominent component of endometriosis, its importance is underexplored, particularly in relation to EMT. ^37, 120^ For early clinical studies of EMT, the nude mouse is a suitable model, particularly for the identification of MMP-2 and TIMP-2, proteins that seem to play a significant role in the pathophysiology of EMT. Estrogen specifically increases MMP-2 expression to encourage ectopic implantation of the endometrium. On the other hand, progestin can suppress TIMP-2 expression, increasing the MMP-2/TIMP-2 ratio and increasing the invasiveness of the ectopic endometrium to facilitate implantation. ^121^ In ovarian endometriosis, MMP7 facilitates EMT; EGF increases MMP7 expression by activating the ERK1–AP1 pathway. ^122, 123^ MMP14 affects the development and function of invadopodia, which in turn modulates the ability of mesenchymal cells to invade and migrate. ^124^ MMP-2 and MMP-9, two important enzymes involved in the destruction of diverse types of ECM, have been linked to the development of endometriosis by regulating endometrial cell invasion. ^125^ Both MMP-2 and MMP-9 have been shown to function as biomarkers of both EMT and triggering factors that contribute to the progression of EMT. ^126^ Despite this, it is apparent that MMPs play crucial roles in the production of collagen, which is necessary for the gradual development of endometriosis fibrosis. ^99^ These findings suggest that there may be a precise equilibrium between collagen synthesis and breakdown, which should be investigated further. As a result, we hypothesize that MMPs may be crucial in controlling the endometriosis-related EMT process. However, further research is needed to fully understand the connection between MMPs and EMT-induced fibrosis in endometriosis, as there are not enough comprehensive studies on this topic.

## Discussion

Endometriosis is an underdiagnosed chronic inflammatory disease that affects millions of people around the world. The primary explanation for endometriosis growth is the transplantation of living endometrial cells that are refluxed after menstruation, thereby attaching to and invading other pelvic organs and leading to inflammation and fibrosis. ^2^ Despite its broad incidence and importance, endometriosis research has significant limitations. ^127^ The gaps include a lack of understanding of the disease’s etiology, a delay in diagnosis that necessitates invasive treatments, and the difficulties of integrating electronic health records for research, which aids in identifying potential therapeutic tools and reminds us to look beyond endometriotic lesions. ^128^ Currently, 50 to 70% of endometriotic drugs that have advanced to phases II and III in clinical trials are unable to show efficacy, suggesting an unfulfilled research gap in the development of appropriate animal models. ^129^ Endometriotic fibrosis shares characteristics with other fibrotic conditions, including increased myofibroblast and smooth muscle cell activity, high levels of fibrotic-associated growth factor and protein production, epithelial–mesenchymal transition, and collagen deposition. ^15^ There is substantial evidence that fibrosis is a molecular characteristic of endometriosis etiology along with other molecular hallmarks, such as immunological dysregulation, ER expression, progesterone resistance, chronic inflammation, angiogenesis, and epigenetic changes. ^15^ Interestingly, fibrosis, as a histologic feature of lesions, can progress, most likely due to repeated tissue injury and repair caused by inflammation-induced recurrent menstrual bleeding. ^47, 127^ Thus, a thorough understanding of the disease process is needed for progress in the fields of biomarker identification and nonhormonal therapy. Fibrosis may impair drug administration and efficacy. Rather, a study into the mechanisms that resolve fibrosis will uncover new possibilities by discovering new targets for pharmacologically regulating this condition, notably in the pharmacology of multicomponent medications. ^128, 130^ Because chronic fibrosis plays vital role in various human body systems, robust longitudinal studies are needed to [a] confirm biomarkers and underlying mechanisms linked with fibrosis progression, providing insights into disease causes and potential diagnostic or prognostic tools. [b] To investigate temporal dynamics to record the progression of fibrosis over time, researchers can better comprehend its development from early stages to advanced stages, thereby allowing early intervention and personalized treatment methods. [c] Investigating treatment efficacy, or the effectiveness of various interventions for fibrosis, can provide useful data on long-term outcomes and responses. [d] To better understand the natural course of fibrosis, including its variations among individuals, potential triggers, and variables influencing its progression, preventive and targeted therapeutics should be created. [e] To determine whether the inflammatory environment of endometriosis is involved in fibrosis. The potential pathways by which endometriosis participates in fibrosis require additional exploration. Indeed, developing fibrosis-specific treatments for endometriosis remains a major challenge. Therefore, drug repurposing could be a viable approach in this quest. Novel anti-fibrotic drug Pirfenidone has been shown to decrease postoperative adhesion formation following laparoscopic endometriosis surgery. ^131^ Considerable research has been made in the specific blockade of cytokines or their downstream signaling pathways for the treatment of fibrotic diseases in general. Nonspecific targeting of S100A4 has been tried using Niclosamide that performs blanket targeting of several signalling pathways, including S100A4, mTOR, STAT3, and NF-κB. ^132^ However, a peptide antagonist of RAGE (ELKVLMEKEL) was developed based on the sequence of the RAGE-binding domain of HMGB1. The antagonist also worked well to prevent interaction between S100A4 and RAGE. ^133^ The antagonist peptide, named RAGE-antagonist peptide (RAP), has been evaluated as an anti-inflammatory drug in various inflammatory diseases. ^134^ In previous studies, RAP has been found to bind to RAGE and reduce signal transduction-mediated RAGE. The efficacy of RAP as an antifibrotic intervention was validated in bleomycin-induced pulmonary fibrosis.

**Figure 2.**
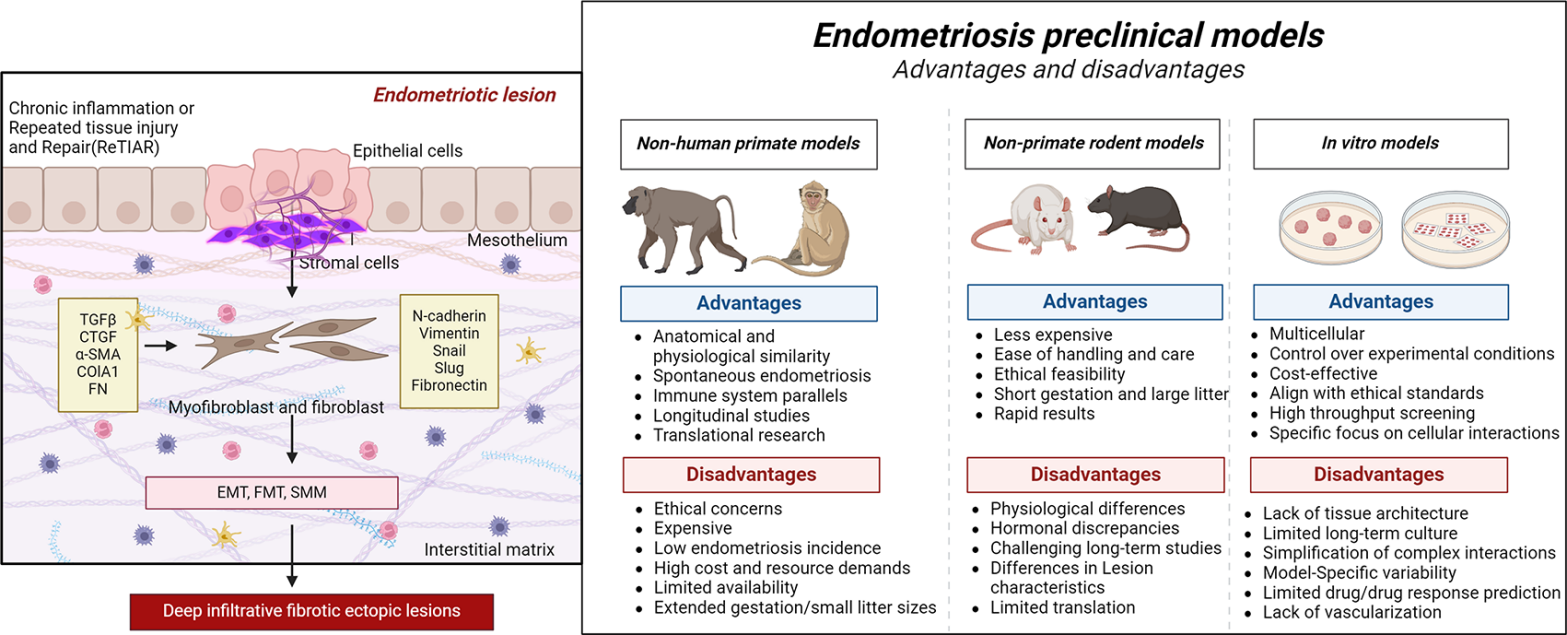
Schematic representation displaying the endometriotic lesion microenvironment and a comparative analysis of nonprimate and nonhuman primate models, emphasizing their advantages and disadvantages in investigating endometriosis. This image shows the importance of selecting appropriate models on the basis of unique research objectives (created with Biorender.com).

Identifying the root cause of endometriosis is more difficult because the disease’s missing components, such as persistent fibrosis, are yet to be duplicated in experimental rodent models. Filling these gaps may lead to more accurate patient diagnoses, more effective treatments, and improved information on how the condition affects women’s lives. Any therapy that helps lessen the fibrotic element of the disease will have far-reaching repercussions for the individual, the population, and the healthcare system. This study contributes to the careful choice of animal models tailored in line with the research objective or study question to improve our understanding of endometriosis ( Figure 2). These findings emphasize the multisystem characteristics of endometriosis, as well as the need for researchers to think beyond only the endometrial lesion. As anticipated, no single cause can entirely explain the onset of endometriosis. However, these investigations emphasize the need for new therapeutic techniques to increase the quality of life of endometriotic patients. The advancement in model development represents a large step forward, delivering promising research with the potential to yield real benefits for patients. Implementing these findings in clinical practice could dramatically shorten diagnostic delays and offer additional insight into the epidemiological elements of the disease.

## Conclusion

Endometriosis is a prevalent gynecological condition that significantly affects the physical and emotional well-being of female patients because of its invasive and recurrent characteristics. Fibrosis, as a histological characteristic of lesions, may progress, presumably due to recurrent tissue injury and repair. In a nonhuman primate model of endometriosis, the predominant type of peritoneal lesion transitioned from red vesicular to white fibrotic over the course of time. However, the association between endometriosis and fibrosis is poorly understood. Additionally, EMT may play a role in the etiology of endometriosis through immunological regulation, the production of proinflammatory cytokines, and other mechanisms. Clinical trials have shown that targeting EMT-induced fibrosis can help treat endometriosis, establishing a new research direction and theoretical foundation for the diagnosis and treatment of fibrotic endometriotic patients. As randomized, double-blinded investigations of endometriosis in women are difficult and at times ethically restrictive, animal models for endometriosis have evolved into vital tools for obtaining a mechanical understanding of the etiology and pathophysiology mechanisms of this complex condition. Thus, it is vital to examine the molecular pathways that drive and sustain fibrosis in endometriosis via a novel fibrosis-based animal model to discover new pharmacological targets and provide creative therapeutics for patients. Furthermore, the research connecting endometriosis and fibrosis has added a further complicating factor to the shared strategy for dealing with endometriotic patients with infertility, as well as a potentially essential concern in the counseling and management of the condition for those desiring future fertility. Well-designed longitudinal studies are needed to improve clinical decision-making in these contexts. Although gynecological surgeons are aware of the complex role of fibrosis in the surgical treatment of endometriosis, the molecular pathways that relate fibrosis to endometriosis-associated pain and infertility remain unknown. Thus, more research is needed to better understand the clinical implications of fibrosis and identify it as a molecular marker of endometriosis etiology, a potentially important element to consider when counseling and managing endometriotic patients who are planning to have children in the future. Well-designed longitudinal studies are needed to make more informed clinical decisions in these contexts. However, the challenges of heterogeneity, diagnostic difficulties, treatment variability, high attrition, and ethical concerns make these studies complex and resource-intensive. Therefore, efforts should be focused on building trustworthy models that incorporate physiologically relevant cells, such as organoids and microfluidics. The continued creation of mouse models to aid in understanding the processes of endometriosis development offers the best chance of creating therapeutic options to prevent or reverse this mysterious disease. This review aims to spark a debate on the need to improve the present understanding by focusing on the fibrotic features of endometriosis pathogenesis. We believe that this approach will shed new light on this condition and suggest areas that need to be investigated further.

## Data Availability

No data are associated with this article.

## References

[1] Bonavina G Taylor HS : Endometriosis-associated infertility: From pathophysiology to tailored treatment. *Front Endocrinol (Lausanne).* 2022;13:1020827. 10.3389/fendo.2022.1020827 36387918 PMC9643365

[2] Sourial S Tempest N Hapangama DK .: Theories on the pathogenesis of endometriosis. Int J Reprod Med. 2014;2014: 179515. 10.1155/2014/179515 PMC4334056 25763392

[3] Gajbhiye RK Montgomery G Pai MV : Protocol for a case-control study investigating the clinical phenotypes and genetic regulation of endometriosis in Indian women: the ECGRI study. *BMJ Open.* 2021 Aug 9;11(8): e050844. 10.1136/bmjopen-2021-050844 34373312 PMC8354274

[4] Yen CF Kim MR Lee CL : Epidemiologic Factors Associated with Endometriosis in East Asia. *Gynecol Minim Invasive Ther.* 2019;8(1):4–11. 10.4103/GMIT.GMIT_83_18 30783582 PMC6367920

[5] Alson S Henic E Jokubkiene L : Endometriosis diagnosed by ultrasound is associated with lower live birth rates in women undergoing their first in vitro fertilization/intracytoplasmic sperm injection treatment. *Fertil Steril.* 2024 May 1;121(5):832–841. 10.1016/j.fertnstert.2024.01.023 38246403

[6] Taylor HS : Endometriosis: a complex systemic disease with multiple manifestations. *Fertil Steril.* 2019 Aug;112(2):235–236. 10.1016/j.fertnstert.2019.06.006 31280952

[7] Gajbhiye RK : Endometriosis and inflammatory immune responses: Indian experience. *Am J Reprod Immunol.* 2023 Feb;89(2): e13590. 10.1111/aji.13590 35751585 PMC7615030

[8] Maddern J Grundy L Castro J : Pain in Endometriosis. *Front Cell Neurosci.* 2020;14: 590823. 10.3389/fncel.2020.590823 33132854 PMC7573391

[9] Sampson JA : Metastatic or Embolic Endometriosis, due to the Menstrual Dissemination of Endometrial Tissue into the Venous Circulation. *Am J Pathol.* 1927 Mar;3(2):93–110.43. 19969738 PMC1931779

[10] Taylor HS Kotlyar AM Flores VA : Endometriosis is a chronic systemic disease: clinical challenges and novel innovations. *Lancet.* 2021 Feb 27;397(10276):839–852. 10.1016/S0140-6736(21)00389-5 33640070

[11] Horne AW Missmer SA : Pathophysiology, diagnosis, and management of endometriosis. *BMJ.* 2022 Nov 14;379: e070750. 10.1136/bmj-2022-070750 36375827

[12] Malvezzi H Marengo EB Podgaec S : Endometriosis: current challenges in modeling a multifactorial disease of unknown etiology. *J Transl Med.* 2020 Aug 12;18(1):311. 10.1186/s12967-020-02471-0 32787880 PMC7425005

[13] Bafort C Beebeejaun Y Tomassetti C : Laparoscopic surgery for endometriosis. *Cochrane Database Syst Rev.* 2020 Oct 23;10(10):CD011031. 10.1002/14651858.CD011031.pub3 33095458 PMC8428328

[14] Garcia Garcia JM Vannuzzi V Donati C : Endometriosis: Cellular and Molecular Mechanisms Leading to Fibrosis. *Reprod Sci.* 2023 May;30(5):1453–1461. 10.1007/s43032-022-01083-x 36289173 PMC10160154

[15] Vigano P Candiani M Monno A : Time to redefine endometriosis including its pro-fibrotic nature. *Hum Reprod.* 2018 Mar 1;33(3):347–352. 10.1093/humrep/dex354 29206943

[16] Yang H Kang K Cheng C : Integrative Analysis Reveals Regulatory Programs in Endometriosis. *Reprod Sci.* 2015 Sep 1;22(9):1060–1072. 10.1177/1933719115592709 26134036 PMC5933170

[17] Ahn SH Monsanto SP Miller C .: Pathophysiology and Immune Dysfunction in Endometriosis. Biomed Res Int. 2015;2015: 795976. 10.1155/2015/795976 PMC4515278 26247027

[18] Herington JL Bruner-Tran KL Lucas JA : Immune interactions in endometriosis. *Expert Rev Clin Immunol.* 2011 Sep;7(5):611–626. 10.1586/eci.11.53 21895474 PMC3204940

[19] Laganà AS Garzon S Franchi M : Translational animal models for endometriosis research: a long and windy road. *Ann Transl Med.* 2018 Nov;6(22):431. 10.21037/atm.2018.08.24 30596061 PMC6281523

[20] Greaves E Critchley HOD Horne AW : Relevant human tissue resources and laboratory models for use in endometriosis research. *Acta Obstet Gynecol Scand.* 2017 Jun;96(6):644–658. 10.1111/aogs.13119 28233896 PMC5485163

[21] Fan H : In-vitro models of human endometriosis. *Exp Ther Med.* 2020 Mar;19(3):1617–1625. 10.3892/etm.2019.8363 32104212 PMC7027135

[22] Bruner-Tran KL Mokshagundam S Herington JL : Rodent Models of Experimental Endometriosis: Identifying Mechanisms of Disease and Therapeutic Targets. *Curr Womens Health Rev.* 2018 Jun;14(2):173–188. 10.2174/1573404813666170921162041 29861705 PMC5925870

[23] D’Hooghe TM Debrock S Hill JA : Endometriosis and subfertility: is the relationship resolved? *Semin Reprod Med.* 2003 May;21(2):243–254. 10.1055/s-2003-41330 12917793

[24] Chiorean DM Mitranovici MI Toru HS : New Insights into Genetics of Endometriosis-A Comprehensive Literature Review. *Diagnostics (Basel).* 2023 Jul 4;13(13):2265. 10.3390/diagnostics13132265 37443659 PMC10340419

[25] Abramiuk M Grywalska E Małkowska P : The Role of the Immune System in the Development of Endometriosis. *Cells.* 2022 Jun 25;11(13):2028. 10.3390/cells11132028 35805112 PMC9265783

[26] Zhang Y Ma NY : Environmental Risk Factors for Endometriosis: An Umbrella Review of a Meta-Analysis of 354 Observational Studies With Over 5 Million Populations. *Front Med (Lausanne).* 2021;8: 680833. 10.3389/fmed.2021.680833 34760897 PMC8573094

[27] Coiplet E Courbiere B Agostini A : Endometriosis and environmental factors: A critical review. *J Gynecol Obstet Hum Reprod.* 2022 Sep;51(7): 102418. 10.1016/j.jogoh.2022.102418 35667590

[28] Zhang Y He T Lin T : Novel in vivo endometriotic models associated eutopic endometrium by implanting menstrual blood-derived stromal cells from patients with endometriosis. *Sci Rep.* 2023 May 23;13(1):8347. 10.1038/s41598-023-35373-4 37221282 PMC10206158

[29] Mori T Ito F Koshiba A : Local estrogen formation and its regulation in endometriosis. *Reprod Med Biol.* 2019 Oct;18(4):305–311. 10.1002/rmb2.12285 31607790 PMC6780031

[30] Laganà AS Garzon S Götte M : The Pathogenesis of Endometriosis: Molecular and Cell Biology Insights. *Int J Mol Sci.* 2019 Nov 10;20(22):5615. 10.3390/ijms20225615 31717614 PMC6888544

[31] Schenken RS Asch RH Williams RF : Etiology of infertility in monkeys with endometriosis: luteinized unruptured follicles, luteal phase defects, pelvic adhesions, and spontaneous abortions. *Fertil Steril.* 1984 Jan 1;41(1):122–130. 10.1016/S0015-0282(16)47552-7 6420199

[32] Hastings JM Fazleabas AT : A baboon model for endometriosis: implications for fertility. *Reprod Biol Endocrinol.* 2006 Oct 9;4(1):S7. 10.1186/1477-7827-4-S1-S7 17118171 PMC1775067

[33] Nishimoto-Kakiuchi A Netsu S Matsuo S : Characteristics of histologically confirmed endometriosis in cynomolgus monkeys. *Hum Reprod.* 2016 Oct;31(10):2352–2359. 10.1093/humrep/dew209 27591226 PMC5027930

[34] Fazleabas A : Models of Endometriosis: Animal Models II - Non-Human Primates. *Endometriosis: Science and Practice.* 2012:285–291. 10.1002/9781444398519.ch27

[35] Cui M Liu Y Men X : Large animal models in the study of gynecological diseases. *Front Cell Dev Biol.* 2023;11:1110551. 10.3389/fcell.2023.1110551 36755972 PMC9899856

[36] Nisenblat V Prentice L Bossuyt PM : Combination of the non-invasive tests for the diagnosis of endometriosis. *Cochrane Database Syst. Rev.* 2016 Jul 13;2016(7):CD012281. 10.1002/14651858.CD012281 PMC6953325 27405583

[37] Somigliana E Vigano P Benaglia L : Adhesion prevention in endometriosis: a neglected critical challenge. *J Minim Invasive Gynecol.* 2012;19(4):415–421. 10.1016/j.jmig.2012.03.004 22575862

[38] Czyzyk A Podfigurna A Szeliga A : Update on endometriosis pathogenesis. *Minerva Ginecol.* 2017 Oct;69(5):447–461. 10.23736/S0026-4784.17.04048-5 28271702

[39] Balasubramanian V Saravanan R Joseph LD : Molecular dysregulations underlying the pathogenesis of endometriosis. *Cell Signal.* 2021 Dec;88: 110139. 10.1016/j.cellsig.2021.110139 34464692

[40] Laudanski P Szamatowicz J Ramel P : Matrix metalloproteinase-13 and membrane type-1 matrix metalloproteinase in peritoneal fluid of women with endometriosis. *Gynecol Endocrinol.* 2005 Aug;21(2):106–110. 10.1080/09513590500154043 16109597

[41] Laudanski P Charkiewicz R Kuzmicki M : Profiling of selected angiogenesis-related genes in proliferative eutopic endometrium of women with endometriosis. *Eur J Obstet Gynecol Reprod Biol.* 2014 Jan;172:85–92. 10.1016/j.ejogrb.2013.10.007 24188612

[42] Koninckx PR Fernandes R Ussia A : Pathogenesis Based Diagnosis and Treatment of Endometriosis. *Front Endocrinol (Lausanne).* 2021;12: 745548. 10.3389/fendo.2021.745548 34899597 PMC8656967

[43] Molina M Moreno GA Singh R : Rectovaginal endometriosis with nodular smooth muscle metaplasia diagnosed via transrectal ultrasound-guided fine-needle aspiration cytology: An underused minimally invasive diagnostic technique? *Diagn Cytopathol.* 2023 Oct;51(10):E273–E278. 10.1002/dc.25183 37318678

[44] Muzii L Bianchi A Bellati F : Histologic analysis of endometriomas: what the surgeon needs to know. *Fertil Steril.* 2007 Feb;87(2):362–366. 10.1016/j.fertnstert.2006.06.055 17094980

[45] Meng XM Nikolic-Paterson DJ Lan HY : TGF-β: the master regulator of fibrosis. *Nat Rev Nephrol.* 2016 Jun;12(6):325–338. 10.1038/nrneph.2016.48 27108839

[46] Scutiero G Iannone P Bernardi G : Oxidative Stress and Endometriosis: A Systematic Review of the Literature. *Oxid Med Cell Longev.* 2017;2017:7265238. 10.1155/2017/7265238 29057034 PMC5625949

[47] Zhang Q Duan J Olson M : Cellular Changes Consistent With Epithelial-Mesenchymal Transition and Fibroblast-to-Myofibroblast Transdifferentiation in the Progression of Experimental Endometriosis in Baboons. *Reprod Sci.* 2016 Oct;23(10):1409–1421. 10.1177/1933719116641763 27076446 PMC5933178

[48] Zhang Q Duan J Liu X : Platelets drive smooth muscle metaplasia and fibrogenesis in endometriosis through epithelial-mesenchymal transition and fibroblast-to-myofibroblast transdifferentiation. *Mol Cell Endocrinol.* 2016 Jun 15;428:1–16. 10.1016/j.mce.2016.03.015 26992563

[49] Asante A Taylor RN : Endometriosis: the role of neuroangiogenesis. *Annu Rev Physiol.* 2011;73:163–182. 10.1146/annurev-physiol-012110-142158 21054165

[50] Mishra A Galvankar M Vaidya S : Mouse model for endometriosis is characterized by proliferation and inflammation but not epithelial-to-mesenchymal transition and fibrosis. *J Biosci.* 2020;45:105. 10.1007/s12038-020-00073-y 32975232

[51] Guo SW Groothuis PG : Is it time for a paradigm shift in drug research and development in endometriosis/adenomyosis? *Hum Reprod Update.* 2018 Sep 1;24(5):577–598. 10.1093/humupd/dmy020 29893860

[52] Perrone U Evangelisti G Laganà AS : A review of phase II and III drugs for the treatment and management of endometriosis. *Expert Opin Emerg Drugs.* 2023 Dec;28(4):333–351. 10.1080/14728214.2023.2296080 38099328

[53] Zondervan KT Weeks DE Colman R : Familial aggregation of endometriosis in a large pedigree of rhesus macaques. *Hum Reprod.* 2004 Feb;19(2):448–455. 10.1093/humrep/deh052 14747196

[54] Dick EJ Hubbard GB Martin LJ : Record review of baboons with histologically confirmed endometriosis in a large established colony. *J Med Primatol.* 2003 Feb;32(1):39–47. 10.1034/j.1600-0684.2003.00008.x 12733601

[55] Nair HB Baker R Owston MA : An efficient model of human endometriosis by induced unopposed estrogenicity in baboons. *Oncotarget.* 2016 Feb 19;7(10):10857–10869. 10.18632/oncotarget.7516 26908459 PMC4905444

[56] Y A, J H, D R, Jw J, Lc G, At F. : Changes in eutopic endometrial gene expression during the progression of experimental endometriosis in the baboon, Papio anubis. *Biol Reprod.* 2013 Feb 21[cited 2024 Nov 16];88(2). 23284138 10.1095/biolreprod.112.104497 PMC3589234

[57] Slayden OD : Induced Endometriosis in Nonhuman Primates. *Biology of Reproduction.* 2013 Jan 9;88(2):43. 10.1095/biolreprod.113.107722 23303683 PMC3589233

[58] D’Hooghe TM Bambra CS Raeymaekers BM : Intrapelvic injection of menstrual endometrium causes endometriosis in baboons (Papio cynocephalus and Papio anubis). *Am J Obstet Gynecol.* 1995 Jul 1;173(1):125–134. 10.1016/0002-9378(95)90180-9 7631669

[59] Kennedy LH Nowland MH Nemzek-Hamlin JA : Surgical treatment of spontaneous endometriosis in rhesus macaques (Macaca mulatta): 11 cases (2007-2011). *J Am Vet Med Assoc.* 2019 Jun 15;254(12):1454–1458. 10.2460/javma.254.12.1454 31149880

[60] Donnez O Van Langendonckt A Defrère S : Induction of endometriotic nodules in an experimental baboon model mimicking human deep nodular lesions. *Fertility and Sterility.* 2013 Mar 1;99(3):783–789.e3. 10.1016/j.fertnstert.2012.10.032 23148925

[61] Harirchian P Gashaw I Lipskind ST : Lesion kinetics in a non-human primate model of endometriosis. *Human Reproduction (Oxford, England).* 2012 Jun 6;27(8):2341–2351. 10.1093/humrep/des196 22674203 PMC3398680

[62] Donnez O Orellana R Kerk OV : Invasion process of induced deep nodular endometriosis in an experimental baboon model: similarities with collective cell migration? *Fertil Steril.* 2015 Aug 1;104(2):491–497.e2. 10.1016/j.fertnstert.2015.05.011 26049053

[63] Orellana R García-Solares J Donnez J : Important role of collective cell migration and nerve fiber density in the development of deep nodular endometriosis. *Fertil Steril.* 2017 Apr 1;107(4):987–995.e5. 10.1016/j.fertnstert.2017.01.005 28238494

[64] Bernal MAO Song Y Joshi N : The Regulation of MicroRNA-21 by Interleukin-6 and Its Role in the Development of Fibrosis in Endometriotic Lesions. *Int J Mol Sci.* 2024 Aug 19;25(16):8994. 10.3390/ijms25168994 39201680 PMC11354763

[65] Dehoux JP Defrère S Squifflet J : Is the baboon model appropriate for endometriosis studies? *Fertil Steril.* 2011 Sep;96(3):728–733.e3. 10.1016/j.fertnstert.2011.06.037 21774926

[66] Zondervan K Cardon L Desrosiers R : The genetic epidemiology of spontaneous endometriosis in the rhesus monkey. Ann N Y Acad Sci. 2002 Mar;955:233–238; discussion 293-295, 396–406. 10.1111/j.1749-6632.2002.tb02784.x 11949951

[67] Wilson RC Link JM Lee YZ : Uterine Uptake of Estrogen and Progestogen-Based Radiotracers in Rhesus Macaques with Endometriosis. *Mol Imaging Biol.* 2024 Apr;26(2):334–343. 10.1007/s11307-023-01892-9 38133866 PMC11034810

[68] Hayashi K Nakayama M Iwatani C : The Natural History of Spontaneously Occurred Endometriosis in Cynomolgus Monkeys by Monthly Follow-Up Laparoscopy for Two Years. *Tohoku J Exp Med.* 2020 Aug;251(4):241–253. 10.1620/tjem.251.241 32713879

[69] Grümmer R : Animal models in endometriosis research. *Hum Reprod Update.* 2006 Sep 1;12(5):641–649. 10.1093/humupd/dml026 16775193

[70] Burns KA Pearson AM Slack JL : Endometriosis in the Mouse: Challenges and Progress Toward a ‘Best Fit’ Murine Model. *Front Physiol.* 2022 Jan 13;12: 806574. 10.3389/fphys.2021.806574 35095566 PMC8794744

[71] Pullen N Birch CL Douglas GJ : The translational challenge in the development of new and effective therapies for endometriosis: a review of confidence from published preclinical efficacy studies. *Hum Reprod Update.* 2011;17(6):791–802. 10.1093/humupd/dmr030 21733981

[72] Dorning A Dhami P Panir K : Bioluminescent imaging in induced mouse models of endometriosis reveals differences in four model variations. *Dis Model Mech.* 2021 Aug;14(8):dmm049070. 10.1242/dmm.049070 34382636 PMC8419713

[73] Fortin M Lépine M Pagé M : An improved mouse model for endometriosis allows noninvasive assessment of lesion implantation and development. *Fertil Steril.* 2003 Sep;80(Suppl 2):832–838. 10.1016/S0015-0282(03)00986-5 14505761

[74] Lee B Du H Taylor HS : Experimental murine endometriosis induces DNA methylation and altered gene expression in eutopic endometrium. *Biol Reprod.* 2009 Jan;80(1):79–85. 10.1095/biolreprod.108.070391 18799756 PMC2804809

[75] Burns KA Rodriguez KF Hewitt SC : Role of estrogen receptor signaling required for endometriosis-like lesion establishment in a mouse model. *Endocrinology.* 2012 Aug;153(8):3960–3971. 10.1210/en.2012-1294 22700766 PMC3404357

[76] Greaves E Cousins FL Murray A : A novel mouse model of endometriosis mimics human phenotype and reveals insights into the inflammatory contribution of shed endometrium. *Am J Pathol.* 2014 Jul;184(7):1930–1939. 10.1016/j.ajpath.2014.03.011 24910298 PMC4076466

[77] Bruner-Tran KL Eisenberg E Yeaman GR : Steroid and cytokine regulation of matrix metalloproteinase expression in endometriosis and the establishment of experimental endometriosis in nude mice. J Clin Endocrinol Metab. 2002 Oct;87(10):4782–4791. 10.1210/jc.2002-020418 12364474

[78] Forster R Sarginson A Velichkova A : Macrophage-derived insulin-like growth factor-1 is a key neurotrophic and nerve-sensitizing factor in pain associated with endometriosis. *FASEB J.* 2019 Oct;33(10):11210–11222. 10.1096/fj.201900797R 31291762 PMC6766660

[79] Wibisono H Nakamura K Taniguchi F : Tracing location by applying Emerald luciferase in an early phase of murine endometriotic lesion formation. *Exp Anim.* 2022 May 20;71(2):184–192. 10.1538/expanim.21-0146 34819403 PMC9130045

[80] García-Gómez E Vázquez-Martínez ER Reyes-Mayoral C : Regulation of Inflammation Pathways and Inflammasome by Sex Steroid Hormones in Endometriosis. *Front Endocrinol (Lausanne).* 2019;10:935. 10.3389/fendo.2019.00935 32063886 PMC7000463

[81] Miller JE Monsanto SP Ahn SH : Interleukin-33 modulates inflammation in endometriosis. *Sci Rep.* 2017 Dec 20;7(1):17903. 10.1038/s41598-017-18224-x 29263351 PMC5738435

[82] Giacomini E Minetto S Li Piani L : Genetics and Inflammation in Endometriosis: Improving Knowledge for Development of New Pharmacological Strategies. *Int J Mol Sci.* 2021 Aug 21;22(16):9033. 10.3390/ijms22169033 34445738 PMC8396487

[83] Cordaro M Trovato Salinaro A Siracusa R : Hidrox® and Endometriosis: Biochemical Evaluation of Oxidative Stress and Pain. *Antioxidants (Basel).* 2021 May 4;10(5):720. 10.3390/antiox10050720 34064310 PMC8147870

[84] Lu H Hu H Yang Y : The inhibition of reactive oxygen species (ROS) by antioxidants inhibits the release of an autophagy marker in ectopic endometrial cells. *Taiwan J Obstet Gynecol.* 2020 Mar;59(2):256–261. 10.1016/j.tjog.2020.01.014 32127147

[85] Tanbo T Fedorcsak P : Endometriosis-associated infertility: aspects of pathophysiological mechanisms and treatment options. *Acta Obstet Gynecol Scand.* 2017 Jun;96(6):659–667. 10.1111/aogs.13082 27998009

[86] Tejada MA Antunez C Nunez-Badinez P : Rodent Animal Models of Endometriosis-Associated Pain: Unmet Needs and Resources Available for Improving Translational Research in Endometriosis. *Int J Mol Sci.* 2023 Jan 26;24(3):2422. 10.3390/ijms24032422 36768741 PMC9917069

[87] Laschke MW Menger MD : Basic mechanisms of vascularization in endometriosis and their clinical implications. *Hum Reprod Update.* 2018 Mar 1;24(2):207–224. 10.1093/humupd/dmy001 29377994

[88] Flores I Rivera E Ruiz LA : Molecular profiling of experimental endometriosis identified gene expression patterns in common with human disease. *Fertil Steril.* 2007 May;87(5):1180–1199. 10.1016/j.fertnstert.2006.07.1550 17478174 PMC1927082

[89] Perrin S : Preclinical research: Make mouse studies work. *Nature.* 2014 Mar 27;507(7493):423–425. 10.1038/507423a 24678540

[90] Umezawa M Sakata C Tanaka N : Cytokine and chemokine expression in a rat endometriosis is similar to that in human endometriosis. *Cytokine.* 2008 Aug;43(2):105–109. 10.1016/j.cyto.2008.04.016 18595729

[91] He Y Liang B Hung SW : Re-evaluation of mouse models of endometriosis for pathological and immunological research. *Front Immunol.* 2022;13: 986202. 10.3389/fimmu.2022.986202 36466829 PMC9716019

[92] Greaves E Rosser M Saunders PTK : Endometriosis-Associated Pain - Do Preclinical Rodent Models Provide a Good Platform for Translation? *Adv Anat Embryol Cell Biol.* 2020;232:25–55. 10.1007/978-3-030-51856-1_3 33278006

[93] Lebman DA Spiegel S : Thematic Review Series: Sphingolipids. Cross-talk at the crossroads of sphingosine-1-phosphate, growth factors, and cytokine signaling. *J Lipid Res.* 2008 Jul;49(7):1388–1394. 10.1194/jlr.R800008-JLR200 18387885 PMC2431110

[94] Chen YJ Li HY Huang CH : Oestrogen-induced epithelial-mesenchymal transition of endometrial epithelial cells contributes to the development of adenomyosis. *J Pathol.* 2010 Nov;222(3):261–270. 10.1002/path.2761 20814901

[95] Yan D Liu X Guo SW : The establishment of a mouse model of deep endometriosis. *Hum Reprod.* 2019 Feb 1;34(2):235–247. 10.1093/humrep/dey361 30561644

[96] Zhang Z Zhou X Xia L : Wenshen Xiaozheng Tang alleviates fibrosis in endometriosis by regulating differentiation and paracrine signaling of endometrium-derived mesenchymal stem cells. *J Ethnopharmacol.* 2025 Jan 10;336: 118724. 10.1016/j.jep.2024.118724 39181283

[97] Matsuzaki S Darcha C : Antifibrotic properties of epigallocatechin-3-gallate in endometriosis. *Hum Reprod.* 2014 Aug;29(8):1677–1687. 10.1093/humrep/deu123 24876174

[98] Wu D Lu P Mi X : Exosomal miR-214 from endometrial stromal cells inhibits endometriosis fibrosis. *Molecular Human Reproduction.* 2018 Jul 1;24(7):357–365. 10.1093/molehr/gay019 29660008

[99] Mohankumar K Li X Sung N : Bis-Indole-Derived Nuclear Receptor 4A1 (NR4A1, Nur77) Ligands as Inhibitors of Endometriosis. *Endocrinology.* 2020 Apr 1;161(4):bqaa027. 10.1210/endocr/bqaa027 32099996 PMC7105386

[100] Guo SW Ding D Geng JG : P-selectin as a potential therapeutic target for endometriosis. *Fertil Steril.* 2015 Apr;103(4):990–1000.e8. 10.1016/j.fertnstert.2015.01.001 25681855

[101] Li J Dai Y Zhu H : Endometriotic mesenchymal stem cells significantly promote fibrogenesis in ovarian endometrioma through the Wnt/β-catenin pathway by paracrine production of TGF-β1 and Wnt1. *Hum Reprod.* 2016 Jun 1;31(6):1224–1235. 10.1093/humrep/dew058 27005891

[102] Hudson N : The missed disease? Endometriosis as an example of ‘undone science.’. *Reprod Biomed Soc Online.* 2021 Aug 13;14:20–27. 10.1016/j.rbms.2021.07.003 34693042 PMC8517707

[103] Simitsidellis I Gibson DA Saunders PTK : Animal models of endometriosis: Replicating the aetiology and symptoms of the human disorder. *Best Pract Res Clin Endocrinol Metab.* 2018 Jun 1;32(3):257–269. 10.1016/j.beem.2018.03.004 29779580

[104] Watson C : Surge in endometriosis research after decades of underfunding could herald new era for women’s health. *Nat Med.* 2024 Feb 1;30(2):315–318. 10.1038/s41591-024-02795-0 38321217

[105] Becker CM Gattrell WT Gude K : Reevaluating response and failure of medical treatment of endometriosis: a systematic review. *Fertil Steril.* 2017 Jul;108(1):125–136. 10.1016/j.fertnstert.2017.05.004 28668150 PMC5494290

[106] Gołąbek-Grenda A Olejnik A : In vitro modeling of endometriosis and endometriotic microenvironment - Challenges and recent advances. *Cell Signal.* 2022 Sep;97: 110375. 10.1016/j.cellsig.2022.110375 35690293

[107] Chen Z Dai Y Dong Z : Co-cultured endometrial stromal cells and peritoneal mesothelial cells for an in vitro model of endometriosis. *Integr Biol.* 2012 Aug 21;4(9):1090–1095. 10.1039/c2ib00172a 22772808

[108] Song Y Burns GW Joshi NR : Endometriotic Organoids as an in vitro Model of Endometriotic Lesion Development [Internet]. *bioRxiv* 2022[cited 2024 Nov 18]. p. 2022.02.15.480583. 10.1101/2022.02.15.480583v1

[109] Shi LB Zhou F Zhu HY : Transforming growth factor beta1 from endometriomas promotes fibrosis in surrounding ovarian tissues via Smad2/3 signaling. *Biol Reprod.* 2017 Dec 1;97(6):873–882. 10.1093/biolre/iox140 29136085

[110] Shao X Wei X : FOXP1 enhances fibrosis via activating Wnt/β-catenin signaling pathway in endometriosis. *Am J Transl Res.* 2018;10(11):3610–3618. 30662612 PMC6291715

[111] Matsuzaki S Darcha C : Co-operation between the AKT and ERK signaling pathways may support growth of deep endometriosis in a fibrotic microenvironment in vitro. *Hum Reprod.* 2015 Jul;30(7):1606–1616. 10.1093/humrep/dev108 25976656

[112] González-Foruria I Santulli P Chouzenoux S : Dysregulation of the ADAM17/Notch signalling pathways in endometriosis: from oxidative stress to fibrosis. *Mol Hum Reprod.* 2017 Jul 1;23(7):488–499. 10.1093/molehr/gax028 28486700

[113] Yan D Liu X Xu H : Mesothelial Cells Participate in Endometriosis Fibrogenesis Through Platelet-Induced Mesothelial-Mesenchymal Transition. *J Clin Endocrinol Metab.* 2020 Nov 1;105(11):e4124–e4147. 10.1210/clinem/dgaa550 32813013

[114] Mihara Y Maekawa R Sato S : An Integrated Genomic Approach Identifies HOXC8 as an Upstream Regulator in Ovarian Endometrioma. *J Clin Endocrinol Metab.* 2020 Dec 1;105(12):e4474–e4489. 10.1210/clinem/dgaa618 32877504

[115] Nagai T Ishida C Nakamura T : Focal Adhesion Kinase-Mediated Sequences, Including Cell Adhesion, Inflammatory Response, and Fibrosis, as a Therapeutic Target in Endometriosis. *Reprod Sci.* 2020 Jul;27(7):1400–1410. 10.1007/s43032-019-00044-1 32329031

[116] Ke J Ye J Li M : The Role of Matrix Metalloproteinases in Endometriosis: A Potential Target. *Biomolecules.* 2021 Nov;11(11):1739. 10.3390/biom11111739 34827737 PMC8615881

[117] Protopapas A Markaki S Mitsis T : Immunohistochemical expression of matrix metalloproteinases, their tissue inhibitors, and cathepsin-D in ovarian endometriosis: correlation with severity of disease. *Fertil Steril.* 2010 Nov;94(6):2470–2472. 10.1016/j.fertnstert.2010.03.007 20385381

[118] Sotnikova NY Antsiferova YS Posiseeva LV : Mechanisms regulating invasiveness and growth of endometriosis lesions in rat experimental model and in humans. *Fertil Steril.* 2010 May 15;93(8):2701–2705. 10.1016/j.fertnstert.2009.11.024 20056200

[119] Rydlova M Holubec L Ludvikova M : Biological activity and clinical implications of the matrix metalloproteinases. *Anticancer Res.* 2008;28(2B):1389–1397. 18505085

[120] Bartley J Jülicher A Hotz B : Epithelial to mesenchymal transition (EMT) seems to be regulated differently in endometriosis and the endometrium. *Arch Gynecol Obstet.* 2014 Apr 1;289(4):871–881. 10.1007/s00404-013-3040-4 24170160

[121] Wang J Ma X : Effects of estrogen and progestin on expression of MMP-2 and TIMP-2 in a nude mouse model of endometriosis. *Clin Exp Obstet Gynecol.* 2012;39(2):229–233. 22905471

[122] Chatterjee K Jana S DasMahapatra P : EGFR-mediated matrix metalloproteinase-7 up-regulation promotes epithelial-mesenchymal transition via ERK1-AP1 axis during ovarian endometriosis progression. *FASEB J.* 2018 Aug;32(8):4560–4572. 10.1096/fj.201701382RR 29558202

[123] Liu F Zhou J Zhang X : Whole-exome sequencing and functional validation reveal a rare missense variant in MMP7 that confers ovarian endometriosis risk. *Hum Mol Genet.* 2022 Aug 17;31(15):2595–2605. 10.1093/hmg/ddac062 35288736

[124] Karamanou K Franchi M Vynios D : Epithelial-to-mesenchymal transition and invadopodia markers in breast cancer: Lumican a key regulator. *Semin Cancer Biol.* 2020 May;62:125–133. 10.1016/j.semcancer.2019.08.003 31401293

[125] Xin L Hou Q Xiong QI : Association between matrix metalloproteinase-2 and matrix metalloproteinase-9 polymorphisms and endometriosis: A systematic review and meta-analysis. *Biomed Rep.* 2015 Jul;3(4):559–565. 10.3892/br.2015.447 26171166 PMC4486806

[126] Orlichenko LS Radisky DC : Matrix metalloproteinases stimulate epithelial-mesenchymal transition during tumor development. *Clin Exp Metastasis.* 2008;25(6):593–600. 10.1007/s10585-008-9143-9 18286378

[127] Ellis K Munro D Clarke J : Endometriosis Is Undervalued: A Call to Action. *Front Glob Womens Health.* 2022 May 10;3: 902371. 10.3389/fgwh.2022.902371 35620300 PMC9127440

[128] Penrod N Okeh C Velez Edwards DR : Leveraging electronic health record data for endometriosis research. *Front Digit Health.* 2023;5:1150687. 10.3389/fdgth.2023.1150687 37342866 PMC10278662

[129] Kimmelman J Federico C : Consider drug efficacy before first-in-human trials. *Nature.* 2017 Jan 30;542(7639):25–27. 10.1038/542025a 28150789

[130] Guo S : Cancer driver mutations in endometriosis: Variations on the major theme of fibrogenesis. *Reprod Med Biol.* 2018 Aug 16;17(4):369–397. 10.1002/rmb2.12221 30377392 PMC6194252

[131] El-Halwagy AS Al-Gergawy AA Dawood AS : Reduction of Postoperative Adhesions after Laparoscopic Surgery for Endometriosis by Using a Novel Anti-Fibrotic Drug Pirfenidone: A Randomized Double Blind Study. *Gynecol Obstet.* 7(1):1–6.

[132] Milani M Mammarella E Rossi S : Targeting S100A4 with niclosamide attenuates inflammatory and profibrotic pathways in models of amyotrophic lateral sclerosis. *J Neuroinflammation.* 2021 Jun 12;18:132. 10.1186/s12974-021-02184-1 34118929 PMC8196441

[133] Hudson BI Lippman ME : Targeting RAGE Signaling in Inflammatory Disease. *Annu Rev Med.* 2018;69(1):349–364. 10.1146/annurev-med-041316-085215 29106804

[134] Piao C Zhuang C Ko MK : Pulmonary delivery of a recombinant RAGE antagonist peptide derived from high-mobility group box-1 in a bleomycin-induced pulmonary fibrosis animal model. *J Drug Target.* 2022 Aug 9;30(7):792–799. 10.1080/1061186X.2022.2069781 35451894

