# Supplementary material for: Unveiling the fibrotic puzzle of endometriosis: An overlooked concern calling for prompt action
Source: F1000Res. 2024 Dec 3;13:721. Originally published 2024 Jul 1. [Version 3] doi: 10.12688/f1000research.152368.3 (PMC11635194; doi:10.12688/f1000research.152368.3)
Supplement: Peer review history

# Reviewer report: Reviewer response for version 3

**DOI:** 10.5256/f1000research.175006.r345346

**Published:** 2024-12-11

**Authors:** Asgerally T Fazleabas

**Affiliations:** 1 Michigan State University, Grand Rapids, MI, USA

The authors have responded appropriately to our concerns. I now approve the manuscript.

Is the review written in accessible language?

Yes

Are all factual statements correct and adequately supported by citations?

Partly

Are the conclusions drawn appropriate in the context of the current research literature?

Partly

Is the topic of the review discussed comprehensively in the context of the current literature?

Partly

Reviewer Expertise:

NA

I confirm that I have read this submission and believe that I have an appropriate level of expertise to confirm that it is of an acceptable scientific standard.

# Reviewer report: Reviewer response for version 2

**DOI:** 10.5256/f1000research.171658.r332392

**Published:** 2024-11-12

**Authors:** Asgerally T Fazleabas, Yong Song

**Affiliations:** 1 Michigan State University, Grand Rapids, MI, USA; 2 Michigan State University, East Lansing, Michigan, USA

This review, titled "Unveiling the Fibrotic Puzzle of Endometriosis: An Overlooked Concern Calling for Prompt Action," addresses the role of fibrosis in endometriosis and highlights the need for improved animal models to study this aspect of the disease. While the topic is important there are significant omissions that do not make this a comprehensive review as the authors claim.

 The specific comments are as follows:

1. In the Introduction: The sentences, “ *Retrograde menstruation is prevalent in healthy women, and only a small population of women develop this condition, contributing to the understanding of complex mechanisms that underlie the onset of this challenging condition. While 90% of women of reproductive age undergo retrograde menstruation to the pelvic cavity, only 10% of them develop endometriosis. These findings suggest that the onset and progression of the disease in the peritoneal cavity depend on additional relevant factors*.” contain duplicative statements. These could be streamlined to improve the clarity.
2. Section “Endometriotic Models: Importance of Addressing Gaps in Preclinical Animal Models”: The preclinical animal models include both rodent and nonhuman primate models, but only rodent models were discussed. Including nonhuman primate models here would provide a more complete overview.
3. Section “Primate Model of Endometriosis”: This section only discusses spontaneous endometriosis. It would be beneficial to include surgically induced endometriosis models in non-human primates, particularly the baboon model, which is valuable for simulating disease establishment and progression. Discussing the surgically induced non-human primate model with respect to fibrosis would add depth to this section.
4. Although the mouse model does not fully capture the fibrosis characteristics seen in human endometriotic lesions, it remains essential for exploring fibrosis’ mechanisms and therapeutic approaches. This model has been valuable for studying the role of inflammation in fibrosis development and developing the methods that detect endometriotic lesions. A more detailed classification of endometriotic fibrotic rodent models in Table 1 would also strengthen this discussion.
5. Section “Human Experiment Details”: The subtitle “Human Experiment Details” is misleading, as this section includes not only human studies but also rodent and non-human primate studies. Consider combining this section with “Interplay of EMT and MMPs in Endometriosis” into a summary of mechanisms contributing to fibrosis in endometriosis. While this section discusses several genes and pathways associated with fibrosis, a more in depth discussion of mechanisms, with specific examples of pathway interactions that promote fibrosis, would enhance the context of the manuscript.
6. The manuscript would benefit from a more detailed comparison of different animal models (non-human primates, mice, rats) and their individual strengths and weaknesses concerning fibrosis research in endometriosis. A table or figure summarizing this information could help readers better understand which models are suitable for addressing specific questions related to this pathology.
7. Table 1 References: Ensure that all references in Table 1 are accurate; for instance, Reference 99 does not involve any animal model.
8. The quality of Figure 2 is not good, and abbreviations should be written out in full in the figure legend for better readability.

Is the review written in accessible language?

Yes

Are all factual statements correct and adequately supported by citations?

Partly

Are the conclusions drawn appropriate in the context of the current research literature?

Partly

Is the topic of the review discussed comprehensively in the context of the current literature?

Partly

Reviewer Expertise:

Endometriosis

We confirm that we have read this submission and believe that we have an appropriate level of expertise to state that we do not consider it to be of an acceptable scientific standard, for reasons outlined above.

# Response

**Published:** 2024-11-18

**Authors:** Rahul Dutta

**Affiliations:** Reproductive Science, Manipal Academy of Higher Education, Manipal, Karnataka, India

Dear Prof Fazleabas

 Accept our gratitude for your detailed critical inputs for improving our manuscript. We have attempted to incorporate most of the suggested changes in the current version of the M/S. We have made changes to the tables and added new figures to improve the quality of review. We enlist the changes made in detail below. We once again, thank you for the opportunity to improve the work,

 Reviewer comment:

1. In the Introduction: The sentences, “Retrograde menstruation is prevalent in healthy women, and only a small population of women develop this condition, contributing to the understanding of complex mechanisms that underlie the onset of this challenging condition. While 90% of women of reproductive age undergo retrograde menstruation to the pelvic cavity, only 10% of them develop endometriosis. These findings suggest that the onset and progression of the disease in the peritoneal cavity depend on additional relevant factors.” contain duplicative statements. These could be streamlined to improve the clarity.

Author response: Despite retrograde menstruation occurring in 90% of reproductive-age women, only 10% develop endometriosis, indicating that additional relevant factors contribute to the disease's onset and progression within the peritoneal cavity. This disparity indicates complex networks contributing to the emergence of this challenging condition.

 Reviewer comment:

1. Section “Endometriotic Models: Importance of Addressing Gaps in Preclinical Animal Models”: The preclinical animal models include both rodent and nonhuman primate models, but only rodent models were discussed. Including nonhuman primate models here would provide a more complete overview.

Author response: The order of sub-sections should be changed here.

- Endometriotic models: Importance of addressing gaps in pre-clinical animal models
- Fibrotic endometriosis overview: knowledge gaps and challenges
- Primate model of endometriosis
- Limitations of non-human primate models in endometriosis research
- Rodent models of endometriosis
- Limitations of rodent models in endometriosis research
- Human experiment details
- Interplay of EMT and MMPs in endometriosis

Changes made:

 Endometriotic models: Importance of addressing gaps in preclinical animal models

 Added at the beginning,

 Due to the unavailability of a definitive treatment and limited understanding of the disease, researchers attempted to develop animal models to provide insights into the disease's causes and to identify novel therapeutic targets. The most extensively studied animal models for endometriosis comprises autologous or syngeneic rodent models, xenotransplantation of human endometrial tissue into immunodeficient mice, and, to a lesser extent due to ethical considerations and expensive costs, nonhuman primate models (19). The most significant distinction between these models is that endometriosis develops spontaneously in non-human primates but not in rodents (19).

 Figure and its legend changed here. (Figure is in PPT).

 Figure 1: Schematic representation of key factors contributing to the development and progression of endometriosis. The illustration highlights the interplay between genetic factors, hormonal imbalances, immune dysfunction, inflammation, including lifestyle related and environmental factors. These factors collectively influence lesion establishment, persistence, growth, providing a comprehensive overview of the multifactorial nature of endometriosis pathophysiology (created with Biorender.com).

 Added primate model details to the same section,

 Taking into account all of these factors as well as possible limitations of rodents, researchers focused on non-human primates like baboons (Papio anubis) and rhesus monkeys because they spontaneously develop endometriosis and menstruate in a cyclic pattern. It's interesting to note that even in nonhuman primates, endometriosis that has been surgically induced reduced fertility, much like it does in humans. In fact, cynomolgus monkeys (Macaca fascicularis) with moderate or severe endometriosis have been shown to have lower rates of fertilization and pregnancy following surgery (31). In addition, subfertility due to endometriosis was tied with stage disease also in baboons (32). The work by Nishimoto-Kakiuchi et al., (33) presents novel and crucial insights from a non-human monkey for translational research in endometriosis where they carefully examined screening, diagnosis, staging, and monitoring in a population of cynomolgus monkeys. They proposed a robust methodology and which has the benefit to employing an animal model with lower body size instead of baboons, making easier for monitoring and handling in an experimental setting. But the major limitation here is the reduced incidence rate of endometriosis of only 28.7% (33). In this context, it appears that non-human primates might serve as the best model organisms in endometriosis research, taking into consideration the similarities to humans regarding phylogenetics, reproductive biology and anatomy, also the presence of spontaneous endometriosis which is identical to its humans (34,35). However, only in some species (Papio anubis) the menstrual periods are nearly every 4 weeks corresponding to that of humans. Indeed, diagnosis of the spontaneous disease in non-human primate models is problematic, as a large number of animals is necessary for the induction and there is a lack of accurate non-invasive tools for the early detection (36). In conclusion, even though non-human primate models are useful for studying the etiology, development, and progression of the disease as well as possibly evaluating the efficacy of drugs, more research is needed to confirm the effectiveness of the "biological response," correlating with endometriosis and its symptoms. This could lead to improved diagnostic accuracy and early detection in non-human primate models, which would be in line with the main goals of clinical endometriosis research in humans.

 This paragraph was deleted. (Owing to differences in opinions concerning the etiology of the disease, the EMT route has received less attention in the context of endometriosis than it does in cancer research. Recently, most research on EMT in endometriosis has focused on tissues; very few studies have examined the specific transcription factors involved in EMT signaling that are present in endometriotic cells 35 , 36 EMT-related processes in endometriosis have been reported to be far more prevalent in ectopic endometrial lesions than in eutopic endometria, suggesting that EMT may contribute to the development of endometriosis. 37 For example, in fibrosis of organs such as the lungs, liver, and kidney, the involvement of the TGF-β signaling pathway is well documented. 38 TGF-β is an influential growth factor and a chemical that attracts monocytes and is capable of triggering fibrosis and angiogenesis during abnormal growth and promoting the progression of endometriosis. 39 Compared with those of normal women, the peritoneal fluid of stage III and IV endometriosis patients has greater levels of TGF-β. 40) and 27 Endometriosis research is mostly based on nonhuman primate or rodent models due to the apparent limitations and ethical concerns of human experimentation. The available mouse models have aided in investigating several aspects of the disorder, such as early disease phases, 41 steroid hormone involvement, 42 host inflammatory mechanisms, 43 , 44 oxidative stress, 45 , 46 neuroangiogenesis, 47 and infertility, 48 in mice.)

 The last paragraph was changed to this.

 In summary, we want to bring attention to the need for an optimal model for understanding endometriosis that mimics the cellular and pathophysiological processes and clinical behaviors observed in human patients, notably fibrosis coupled with invasion and metastasis. Despite these limitations, considerable improvements have been made in the development of endometriotic models for fibrosis-based research studies.

 Reviewer comment:

1. Section “Primate Model of Endometriosis”: This section only discusses spontaneous endometriosis. It would be beneficial to include surgically induced endometriosis models in non-human primates, particularly the baboon model, which is valuable for simulating disease establishment and progression. Discussing the surgically induced non-human primate model with respect to fibrosis would add depth to this section.

 Author response:

 Primate model of endometriosis and limitations (All the references are tagged in the original draft)

 (Added this at the beginning). Endometriosis is challenging to eliminate due to the inadequate understanding of its genesis and pathophysiology. It is recognized to occur exclusively in menstrual animals, including nonhuman primates, such as rhesus macaques (Zondervan et al., 2014) and baboons (Dick et al., 2003), and has demonstrated significant relevance in the study of endometriosis. Because they undergo menstruation, they provide a phylogenetically similar model organism to humans. Their identical endometrial morphology, physiology, and menstrual cycle nearly identical to those of women (Dick et al., 2003). Baboons can also develop spontaneous endometriosis, which makes them one of the most suited and relevant models for investigating this disease (Nair et al., 2016)…..

 ..…The cynomolgus monkey (71,72) has been described, with the limitations that deep lesions were difficult to diagnose and time course changes in the condition were not investigated. (Add the following details after this sentence). Two types of endometriotic models have been established in baboons: spontaneous (Fazleabas et al., 2002) and experimentally generated endometriosis via autologous endometrial transplantation (Afshar et al., 2013, Slayden, O. D. 2013) Moreover, induced endometriosis in nonhuman primates demonstrated has been shown to closely resemble spontaneous endometriosis developing in women (D'Hooghe et al., 1995). It was also claimed that iatrogenically induced retrograde menstruation might lead to the onset of endometriosis validating the concept of Sampson. In fact, endometriosis was experimentally generated in rhesus macaques via surgical diversion of cervix into the abdomen. Yet, endometriosis was identified in only 50% of the animals (Kennedy et al., 2019). The first baboon experimental model of nodular endometriosis was established in by Donnez et al., 2023 for the exploration of deeper nodular lesions as well as invasion events connected with nodular lesions (Donnez et al., 2013). Frequent surgical interventions, however, are shown to provoke the spontaneous growth of endometriotic lesions and could possibly modify the functionality of the endometrium (Harirchian et al., 2012).

 Regarding the fibrosis aspect of the disease, According to Zhang et al., a baboon endometriosis model demonstrated the progressive nature of EMT, FMT, and fibrosis. This led to the expansion of fibrosis from a minor fibrosis at three months to a highly fibrotic lesion at twelve months after endometriosis induction. This strongly suggests the progressive nature of the disease (Zhang et al., 2016b). Additionally, histological analyses reveal that fibrosis in baboon endometriosis closely mirrors that seen in human cases, making it an appropriate model for investigating disease progression and treatment influences on fibrosis (Giudice et al., 2012). Donnez et al., discovered altered morphology, elevated mitotic activity, and fewer adhesion molecules in invasive glands associated with induced nodular endometriosis implying that cell migration is involved in the process of invasion of deep fibrotic endometriotic lesions generated in a baboon model (Donnez et al., 2015). A model of iatrogenic deep nodular endometriotic lesions was developed in order to build an experimental model of replicating human deep nodular fibrotic lesions (Donnez et al., 2013). Deep nodular endometriotic lesions created in the baboon were shown to closely mirror spontaneous deep-infiltrating nodules in invasive and non-invasive lesions (Donnez et al., 2013, Orellana et al., 2017). A recent investigation in baboon models has increased the understanding of fibrosis in endometriosis. The study indicated the overexpression by IL-6 enhance the expression of fibrotic factors, inducing fibrosis via the TGF-β signalling pathway. These findings in baboons, which closely match human endometriosis, reinforce the concept that fibrosis is a critical component of the disease's course (Ochoa Bernal et al., 2024).

 Limitations of non-human primate models in endometriosis research

 The use non-human primates in endometriosis research has potential drawbacks or limitations. First. Firstly, the low incidence rate i.e., 4.8% and 20.7% of spontaneous and induced endometriosis, respectively demonstrating that baboons are able to cleanse and regenerate their peritoneum which may downgrade the significance of model (Dehoux et al., 2011). Other challenges include relatively small cohort of endometriotic animals for experimentation, difficulty of dealing with conscious baboons, and the high cost of experimentation and maintenance which require larger doses of medications, specialized infrastructure, logistics, and special training for handling these animals. It is also perceived to be ethically sensitive and expensive (Grummer, R. 2006, Slayden, O. D. 2013). Consequently, rodent models are commonly used for preclinical efficacy testing for therapeutic interventions due to their reduced costs and ease of handling.

 Reviewer comment:

1. Although the mouse model does not fully capture the fibrosis characteristics seen in human endometriotic lesions, it remains essential for exploring fibrosis’ mechanisms and therapeutic approaches. This model has been valuable for studying the role of inflammation in fibrosis development and developing the methods that detect endometriotic lesions. A more detailed classification of endometriotic fibrotic rodent models in Table 1 would also strengthen this discussion.

 Author response: 

 (Major changes done in this section).

 Preclinical modeling is crucial for investigations of disease pathogenesis, biomarker development, and preventative and therapeutic discovery. This is particularly true for complex conditions, such as endometriosis where non-surgical diagnostic techniques to allow longitudinal clinical study designs remain unavailable. Rodents are frequently employed as a preclinical model in biomedical research since they are a molecularly well-annotated species. This permits researchers to utilize different interrogative strategies to dissect multifactorial disorders. Their usefulness for examining the molecular foundations of disease pathogenesis lies in the simplicity of genetic modifications and their ability to target potential genes for specialized study (70). Additionally, given the lack of accessibility and high costs related to non-human primates, rodents offer as a convenient and inexpensive alternative for researching the origins and course of disorders like endometriosis. However, because research facilities for primates/non-human are constrained, non-primate experimental animal species, such as mice or rats, are regarded suitable first-line tools for researching the origin of this puzzling disease. Endometriosis is characterized by the recurrent development of new lesions with each menstrual cycle and the advancement of preexisting lesions. Therefore, additional research is needed to understand the natural course and gradual development of endometriosis lesions (71). There is evidence of gradual lesion clearing, but only a small number of studies using mouse models of endometriosis have investigated disease induction and regression (71,72). While rodent models have been valuable for researching the disease, especially its pathophysiological and molecular underpinnings, gaps exist in understanding fibrotic lesion progression. Most importantly, due to the ethical limits of frequent laparoscopic screening of endometriotic patients, rodent models provide essential longitudinal investigations to boost the translational value of preclinical findings (71).

 Mice are the most popular experimental animal models because of their ease of gene manipulation, availability, easy handling, tissue similarity in vivo, small size and large litter, which make them cost-effective, and their relatively short gestation, which allows transgenerational examination (22). Based on the available research publications, two types of mouse models have been successfully used to implant endometriotic lesions. The first approach involves suturing, where human endometriotic implants are surgically auto-transplanted into the peritoneum of immunocompromised mice (73–75). The second approach involves the intraperitoneal or subcutaneous implantation of autologous uterine segments into the peritoneum of recipient mice from a syngeneic donor (76–78). The mouse models have aided in investigating several aspects of the disorder, such as early disease phases (79) steroid hormone involvement (80), host inflammatory mechanisms (81,82), oxidative stress (83,84), neuroangiogenesis (76), and infertility (85).While these methods have enhanced our understanding of disease pathways, challenges persist. For example, Immunocompetence is a difficulty when employing human uterine tissue or human endometriotic tissue in a mouse model. Immunocompromised mice may not reflect the environment within the human peritoneal cavity, and the outcomes of the experiment may not correctly reflect disease onset (86). In ovariectomized mice models using exogenous estrogen, it was proven that estrogen reliance drives lesion progression in endometriosis; However, these models added surgical factors and off-target effects. Because endometriosis mirror natural hormonal cycles, hormonally intact mice offer a more realistic representation (75). However, mouse like other members of the rodent family, is typically do not menstruate and hence does not develop endometriosis spontaneously. They also have a closed reproductive system and are highly fragile with dietary needs. Consequently, earlier studies modeling endometriosis utilizing mice required stimulation of menstruation or endometrium transplantation for the development of endometriotic lesions (70). Hence, there are publications which claim that these lesions do not adequately mirror real endometriosis as they lack features such as persistent fibrosis (87).

 On the other hand, rats can only produce superficial lesions, which are the most fundamental and possibly least clinically significant types of lesions. Many studies using rodents as a model for endometriosis have investigated the gene expression patterns of ectopic tissue deposits in rats in an attempt to correlate them with human endometriotic lesions. Chronic inflammation, angiogenesis, and extracellular matrix remodeling are common pathways (86–88). While some aspects of the disease are replicated in the rodent model, all the modifications involve suturing uterine fragments (endometrium plus myometrium) to different sites, which does not accurately represent the formation of lesions from those shed endometrial tissue or the dissemination of menstrual tissue into the peritoneum. Notably, particularly in terms of understanding its pathophysiology and treatment options, the current rodent models have not been successful in yielding findings that apply to human endometriosis. The inability of any study to recreate fibrotic endometriotic lesions may account for the failure of rat models to yield data relevant to the pathophysiology and treatment of human endometriosis. This situation demonstrates that the preclinical animal studies that have been established are not transferable (91).Therefore, fibrosis, a mostly disregarded component of human endometriosis, should be taken into consideration (90, 91). We reviewed the existing mouse models in the context of optimal parameters found on well-evidenced pathophysiologic aspects identified in endometriosis (Table 1). Collectively, these models have yielded critical insights and sustained advancement toward replicating the molecular characteristics of this disease. With completing knowledge gaps such as the modeling of chronic fibrosis, mouse models constitute a powerful resource for translational research in endometriosis. Therefore, developing novel rodent models that mirror the continuous fibrotic process observed in endometriotic patients is essential for improving our understanding of this disease. Emerging research has recently focused on the role that fibrosis plays in clinical-grade endometriosis. On the other hand, little is known about fibrosis treatment strategies. Therefore, developing a fibrotic mouse model of endometriosis, elucidating the regulatory processes underlying fibrosis in endometriosis, and identifying more precise specific biomarkers for this disease are critical. These markers can also be utilized to find effective therapeutic targets and identify endometriosis in its early phases. The successful translation of potential discoveries obtained in a preclinical model to human is primarily dependent on model fidelity. To mimic the fibrotic scarring observed in endometriosis, many endometriotic fibrotic animal models have been developed (Table 1).

 (Deleted this). To mimic the fibrotic scarring observed in endometriosis, many endometriotic fibrotic animal models have been developed ( Table 1). Furthermore, new in vivo models that use stromal cells generated from menstrual blood have been created to study endometriosis; these models show enhanced endometriotic cell migration and proliferation. 58 Many cues, including estrogen stimulation, may trigger EMT. 88 Furthermore, estrogen-induced EMT in Ishikawa cells promotes adenomyosis. 89 However, how estrogen causes EMT in endometriosis at the molecular level remains unknown. To prevent fluctuations in mouse estradiol levels during the estrous cycle, the majority of established mouse models use ovariectomized mice. 90– 93 As a result, the steady availability of estradiol in the circulation may help promote lesion establishment and growth. However, research on how estrogen-induced EMT in endometriosis affects fertility, such as in women with normal circulating estrogen, is impossible. Therefore, studies of endometriosis produced in intact mice call for more research on the connection between ectopic tissue and fertility.

 These findings suggest potential targets for treatment to mitigate fibrosis. (Change this paragraph). Many of the current animal models of endometriosis can be further enhanced by altering them to allow non‐invasive in vivo monitoring of lesion size as it is desirable for preclinical models of endometriosis. Although, studies have given insight on identifying genes that contribute to fibrosis in endometriosis, more exploration of the complicated signaling networks underlying the disease remains important. This gap points out the necessity for future investigations employing advanced methodologies such as knockout animal models, high-throughput RNA sequencing, and omics techniques. These techniques provide greater insights into the mechanisms of fibrotic markers and assist in confirming their function in endometriosis growth, providing strong evidence for the creation of medications that delay, terminate, and reverse fibrosis advancement and benefit endometriotic patients.

 Table and the legend has been changed.

 Table 1: Summary of available mice models of endometriosis, demonstrating the presence of fibrotic markers. The table includes details on the type of model, approach used for the model development, and the specific fibrotic markers and the pathways explored. This analysis emphasizes the heterogeneity in fibrotic marker expression across different models and provides insights into their relevance for researching the fibrotic elements of endometriosis. Alpha-smooth muscle actin (α-SMA), connective tissue growth factor (CTGF), Fibronectin (FN), Transforming Growth Factor Beta (TGF-β), COL1A1 and A3 (Collagen type 1 and 3), epithelial-to-mesenchymal transition (EMT), fibroblast-to-myofibroblast transdifferentiation (FMT), and smooth muscle metaplasia (SMM), tumor necrosis factor α (TNFα) and the monocyte chemoattractant proteins chemokine ligands 2 and 5, Fibroblast-Specific Protein 1 (FSP1), S100 Calcium Binding Protein A4 (S100A4), TNF-α (Tumor Necrosis Factor-alpha) and IL-6 (Interleukin-6), Mesothelial-mesenchymal transition (MMT), and Endothelial-mesenchymal transition (EndoMT), Endometrium-derived mesenchymal stem cells (eMSCs), mammalian target of rapamycin (mTOR)

 Added limitations

 Limitations of rodent models in endometriosis research

 Endometriosis is termed the ‘missing disease’ because of its ambiguous etiology and discrepancies in its origin, diagnosis and treatment (103). Despite a recent surge in endometriosis research, the underlying pathobiology of the disease remains poorly known, implying that animal models of the disorder are crucial for future studies in this field. This ambiguity highlights the need for animal models that precisely mimic human endometriosis and elucidate its conditions, which can provide a basis for subsequent research (104). One of the most significant obstacles in endometriosis research is the lack of reliable mouse models that characterize the manifestations of this condition in humans (105). Ideally, a disease model should mirror human disease while also allowing researchers to investigate the effects of intrinsic (e.g., genes) and extrinsic (e.g., environment) factors on disease progression. Many previous studies linked fibrosis secondary to the development of endometriosis, and there has not been much research on fibrosis itself (15,106). Research from animal models clearly revealed that a percentage of women receiving hormone therapy in human trials do not respond to these drugs (106) and require surgical lesion removal to alleviate symptoms. Women may have endometriotic lesions that have progressed to a fibrotic state by the time they seek medical attention, rendering treatment ineffective. This highlights the urgent need for the establishment of an in vivo model that can effectively mimic the development and characteristics of human endometriosis, opening avenues for more effective treatments and a deeper understanding of this disease. These findings will also facilitate the understanding of the connection between the origin of fibrosis in endometriosis, existing medical care, and potential targets for therapy. In conclusion, although literature emphasizes the significance of fibrosis in the course of endometriosis, there remain gaps in understanding the underlying genes and pathways related to the fibrotic aspect of the disease. While the existing rodent models highlight certain factors such as inflammation and immune dysregulation, they often overlook fibrosis, thus poorly reflecting the complexity of the disease. In addition, these models insufficiently depict the degree of severity, traits, and drivers of fibrosis in clinical human endometriosis. Also, the complex interplay of signalling mechanisms that promote lesion formation in a fibrotic milieu remain inadequately studied. These limitations highlight the demand for improved fibrotic based animal models that accurately replicate the disease which offer an in-depth investigation of fibrotic pathways. Although, studies have given insight on identifying genes that contribute to fibrosis in endometriosis, more exploration of the complicated signaling networks underlying the disease remains important. This gap points out the necessity for future investigations employing advanced methodologies such as knockout animal models, high-throughput RNA sequencing, and omics techniques. These techniques provide greater insights into the mechanisms of fibrotic markers and assist in confirming their function in endometriosis growth, providing strong evidence for the creation of medications that delay, terminate, and reverse fibrosis advancement and benefit endometriotic patients. Also, many of the current animal models of endometriosis can be further enhanced by altering them to allow non‐invasive in vivo monitoring of lesion size as it is desirable for preclinical models of endometriosis.

 Reviewer comment:

1. Section “Human Experiment Details”: The subtitle “Human Experiment Details” is misleading, as this section includes not only human studies but also rodent and non-human primate studies. Consider combining this section with “Interplay of EMT and MMPs in Endometriosis” into a summary of mechanisms contributing to fibrosis in endometriosis. While this section discusses several genes and pathways associated with fibrosis, a more in depth discussion of mechanisms, with specific examples of pathway interactions that promote fibrosis, would enhance the context of the manuscript.

Author response:

 The content under human experiment details is completely changed.

 Except for several non-human primates, animals do not develop endometriosis spontaneously and hence in vitro models employing human tissues have been employed to research the pathophysiology of this medical condition (Table 2). The majority of currently known in vitro models utilize a number of cell or tissues types, including endometriotic cell lines as the monolayer culture model, human primary endometrial epithelial and stromal cells, endometrial stem cells, endometrial explant culture, co-culture models with peritoneal cells and immune cells (107–109). Each model exhibits unique characteristics and functions and were able to illustrate one or more components of the process of endometriosis. These models are helpful and can be used to explore the origin of endometriosis and the underlying mechanisms of this condition in depth, and assist investigators select relevant models for their research (21). In recent years, researchers developed different in vitro models of varying complexity that provide helpful tools to unravel processes involved in the etiology of endometriosis. Most cell culture methods are maintained in 2D settings; however, more advanced 3D models are becoming more prevalent to better the specific endometriosis milieu. They offer the chance to examine endometriotic cell connection with surrounding cells and analyze unique cross-talks between cells (107). The patient obtained tissues of ectopic and eutopic endometrium or biopsy samples from the endometriotic cysts and fluids from women with and without endometriosis undergoing laparoscopy for diverse research goals are being used. However, protocol variation employed for collecting, processing, and storing samples certainly restricts the compilation and repeatability of data produced in different research institutions

 According to Fan 2020, except for studying the origin and mechanisms behind fibrosis in endometriosis, in vitro models are a viable tool to investigate therapeutic innovations for the management of endometriosis (21). The idea that endometriosis is a fibrotic disease has prompted studies to look into how myofibroblasts differentiation and how fibrosis develops in endometriotic lesions. This will lead to the development of new models that can be used to study endometriotic fibrosis. Thus, future studies should concentrate on the myofibroblasts differentiation and activity in endometriotic lesions. Advances in modeling in vitro technology could potentially revolutionize the study of endometriosis pathophysiology and allow the discovery of new targets to develop effective treatment approaches.

 New table has been added and legend has been changed

 Table 2: Overview of in vitro studies on endometriosis tissues demonstrating the presence of fibrotic markers. The table outlines the type of endometriosis tissue used, specific fibrotic markers evaluated, and key pathways. This compilation highlights the contributions of in vitro systems in unraveling the molecular mechanisms underlying fibrosis in endometriosis. OE- Ovarian endometrioma, DIE-Deep infiltrating endometriosis, Transforming Growth Factor β1 (TGF-β1) Pathway, Platelet-Derived Growth Factor (PDGF) Pathway, Wnt/β-catenin Pathway, α-SMA (alpha-Smooth Muscle Actin), COL1A1 (Collagen Type I Alpha 1 Chain), CTGF (Connective Tissue Growth Factor), FN (Fibronectin), rapamycin (mTOR) signaling, GLI3: GLI Family Zinc Finger 3, HOXC8, HOXA9 and A10: Homeobox C8 and A10, MAPK8: Mitogen-Activated Protein Kinase 8 (also known as JNK1), ETS2: ETS Proto-Oncogene 2, Transcription Factor, GATA2: GATA Binding Protein 2, FAK (Focal Adhesion Kinase), TFAP2C: Transcription Factor AP-2 Gamma, PRDM1: PR/SET Domain 1 (also known as BLIMP-1)

 Reviewer comment:

1. The manuscript would benefit from a more detailed comparison of different animal models (non-human primates, mice, rats) and their individual strengths and weaknesses concerning fibrosis research in endometriosis. A table or figure summarizing this information could help readers better understand which models are suitable for addressing specific questions related to this pathology.

Author response:

 Limitations was discussed in corresponding section

 Figure 2 has been added for the comparison of primate, rodent and in vitro as suggested

 Figure 2: Schematic representation displaying the endometriotic lesion microenvironment and a comparative analysis of non-primate and non-human primate models, emphasizing their advantages and disadvantages in investigating endometriosis. This image shows the importance of selecting appropriate models based on unique research objectives (Created with Biorender.com).

 Reviewer comment:

1. Table 1 References: Ensure that all references in Table 1 are accurate; for instance, Reference 99 does not involve any animal model.

Author response:

 Changed Table 1 with new references

 Reviewer comment:

1. The quality of Figure 2 is not good, and abbreviations should be written out in full in the figure legend for better readability.

Author response:

 Changed Figure 2

# Reviewer report: Reviewer response for version 2

**DOI:** 10.5256/f1000research.171658.r328656

**Published:** 2024-10-12

**Authors:** Jae-Wook Jeong, Md Saidur Rahman

**Affiliations:** 1 University of Missouri, Columbia, Missouri, USA

The authors have adequately addressed my concerns and the paper is improved accordingly.

Is the review written in accessible language?

Yes

Are all factual statements correct and adequately supported by citations?

Partly

Are the conclusions drawn appropriate in the context of the current research literature?

Partly

Is the topic of the review discussed comprehensively in the context of the current literature?

Partly

Reviewer Expertise:

Endometriosis; Translational study; Preclinical animal model

We confirm that we have read this submission and believe that we have an appropriate level of expertise to confirm that it is of an acceptable scientific standard.

# Response

**Published:** 2024-10-12

**Authors:** Rahul Dutta

**Affiliations:** Reproductive Science, Manipal Academy of Higher Education, Manipal, Karnataka, India

We are thankful to the reviewers for their constructive input. We thank the reviewers for approving the revised version of the manuscript.

# Reviewer report: Reviewer response for version 1

**DOI:** 10.5256/f1000research.167117.r311329

**Published:** 2024-08-22

**Authors:** Jae-Wook Jeong, Md Saidur Rahman

**Affiliations:** 1 University of Missouri, Columbia, Missouri, USA

Overall Rating: Average

 Reviewer Opinion: Major Revision

 The manuscript effectively emphasizes the significance of understanding endometriosis-related fibrosis and the limitations of current treatments. It underscores the need for better models and addresses the challenges in understanding the disease's pathogenesis. However, the presentation would benefit from a more structured approach and a clearer focus on specific research gaps and proposed solutions. Adding details on how the review addresses these gaps and improves therapeutic strategies would enhance its impact. While the review offers valuable insights into fibrotic models and treatments, greater clarity on its specific contributions is needed.

  

- Abstract: The abstract effectively outlines the importance of fibrosis research in endometriosis. Restructuring for clarity and adding specific research gaps and proposed solutions would strengthen overall outlook.
- Introduction:
  - Link to Fibrosis: The introduction does not clearly connect endometriosis issues to fibrosis. Adding specifics on how the review addresses this would improve its rationale.
  - Redundancy: The introduction repeats information on endometriosis mechanisms and diagnostic challenges. Reducing repetition would improve clarity.
- Literature Review:
  - Fibrotic Endometriosis Overview: The discussion on animal model limitations could be more focused. Highlighting specific shortcomings, especially in fibrosis and EMT representation, would provide clearer insights. There is a mouse model for endometriosis and fibrosis (PMID: 30626716).
  - Endometriotic Models: While the challenges of developing accurate models are discussed, more specific examples would be beneficial. Consider adding key limitations of rodent models and clarifying how estrogen-induced EMT impacts translation to human disease.
  - EMT and MMPs in Endometriosis: The discussion on MMPs and EMT lacks detailed mechanisms and direct evidence. Emphasizing experimental findings and strengthening the link between MMPs, EMT, and disease progression would improve this section.
- Discussion: While acknowledging the importance of translating research into clinical care, the section lacks concrete examples of how this might occur or what specific therapeutic advancements are expected.
- Conclusion: The call for longitudinal studies is important, but the section does not address the obstacles in conducting these studies.

Minor

 Abstract: the first sentence: “estrogen-dependent” repeated word

 mesenchymal to epithelial transition (FMT) change to “MET”

Is the review written in accessible language?

Yes

Are all factual statements correct and adequately supported by citations?

Partly

Are the conclusions drawn appropriate in the context of the current research literature?

Partly

Is the topic of the review discussed comprehensively in the context of the current literature?

Partly

Reviewer Expertise:

Endometriosis; Translational study; Preclinical animal model

We confirm that we have read this submission and believe that we have an appropriate level of expertise to state that we do not consider it to be of an acceptable scientific standard, for reasons outlined above.

# Response

**Published:** 2024-09-16

**Authors:** Rahul Dutta

**Affiliations:** Reproductive Science, Manipal Academy of Higher Education, Manipal, Karnataka, India

Dear Mr. Reviewer,

 We are thankful to you for the constructive feedback.  We have incorporated the suggested improvement into the revised manuscript.   

 Here are the specific responses to the comments/suggestions-

**Abstract: The abstract effectively outlines the importance of fibrosis research in endometriosis. Restructuring for clarity and adding specific research gaps and proposed solutions would strengthen overall outlook.**

*The abstract has been modified as advised*

 Introduction:

**Link to Fibrosis: The introduction does not clearly connect endometriosis issues to fibrosis. Adding specifics on how the review addresses this would improve its rationale.-**

*The introduction has been modified as advised*

**Redundancy: The introduction repeats information on endometriosis mechanisms and diagnostic challenges. Reducing repetition would improve clarity.**

*The redundant portion has been edited as advised*

**Minor Abstract: the first sentence: “estrogen-dependent” repeated word**- *Removed*

**mesenchymal to epithelial transition (FMT) change to “MET”-** *Changed*

 Literature Review: 

**Fibrotic Endometriosis Overview: The discussion on animal model limitations could be more focused. Highlighting specific shortcomings, especially in fibrosis and EMT representation, would provide clearer insights.**

*The overview has been modified as advised*

**There is a mouse model for endometriosis and fibrosis (PMID: 30626716).**

*But it is developed in Baboon, what we are trying to discuss here is the fibrotic mice model*

**Endometriotic Models: While the challenges of developing accurate models are discussed, more specific examples would be beneficial. Consider adding key limitations of rodent models and clarifying how estrogen-induced EMT impacts translation to human disease.**

*Modified as advised*

**EMT and MMPs in Endometriosis: The discussion on MMPs and EMT lacks detailed mechanisms and direct evidence. Emphasizing experimental findings and strengthening the link between MMPs, EMT, and disease progression would improve this section.**

*Modified as advised*

*We extend our heartfelt gratitude for the feedback.*
